# Effect of M6A regulators on diagnosis, subtype classification, prognosis and novel therapeutic target development of idiopathic pulmonary fibrosis

**DOI:** 10.3389/fphar.2022.993567

**Published:** 2022-11-28

**Authors:** Guirui Huang, Shuaiyang Huang, Hongsheng Cui

**Affiliations:** Department of Respiratory, The Third Affiliated Hospital, Beijing University of Chinese Medicine, Beijing, China

**Keywords:** idiopathic pulmonary fibrosis, m6A regulators, diagnostic model, consensus clustering, prognostic markers, novel therapeutic targets

## Abstract

Molecular biology studies show that RNA N6-methyladenosine (m6A) modifications may take part in the incidence and development of idiopathic pulmonary fibrosis (IPF). Nonetheless, the roles of m6A regulators in IPF are not fully demonstrated. In this study, 12 significant m6A regulators were filtered out between healthy controls and IPF patients using GSE38958 dataset. Random forest algorithm was used to identify 11 candidate m6A regulators to predict the incidence of IPF. The 11 candidate m6A regulators included leucine-rich PPR motif-containing protein (LRPPRC), methyltransferase-like protein 3, FTO alpha-ketoglutarate dependent dioxygenase (FTO), methyltransferase-like 14/16, zinc finger CCCH domain-containing protein 13, protein virilizer homolog, Cbl proto-oncogene like 1, fragile X messenger ribonucleoprotein 1 and YTH domain containing 1/2. A nomogram model was constructed based on 11 candidate m6A regulators and considered beneficial to IPF patients using decision curve analysis. Consensus clustering method was used to distinctly divide IPF patients into two m6A patterns (clusterA and clusterB) based on 12 significant m6A regulators. M6A scores of all IPF patients were obtained using principal component analysis to quantify the m6A patterns. Patients in clusterB had higher m6A scores than those in clusterA. Furthermore, patients in clusterB were correlated with Th17 and Treg cell infiltration, innate immunity and Th1 immunity, while those in clusterA were correlated with adaptive immunity and Th2 immunity. Patients in clusterB also had higher expressions of mesenchymal markers and regulatory factors of fibrosis but lower expressions of epithelial markers. Lastly and interestingly, two m6A regulators, LRPPRC (*p* = 0.011) and FTO (*p* = 0.042), were identified as novel prognostic genes in IPF patients for the first time using an external GSE93606 dataset. Both of them had a positive correlation with a better prognosis and may serve as therapy targets. Thus, we conducted virtual screening to discover potential drugs targeting LRPPRC and FTO in the treatment of IPF. In conclusion, m6A regulators are crucial to the onset, development and prognosis of IPF. Our study on m6A patterns may provide clues for clinical diagnosis, prognosis and targeted therapeutic drugs development for IPF.

## Introduction

Idiopathic pulmonary fibrosis (IPF), a chronic interstitial lung disease of unknown causes, is characterized by diffuse alveolitis, epithelial mesenchymal transformation (EMT), and disruption of alveolar structure (Richeldi, Collard et al., 2017; Phan, Paliogiannis et al., 2021). The clinical presentation of IPF is progressive dyspnoea with an irritating dry cough, which usually continues to deteriorate and eventually leads to death from respiratory failure (Moss, Ryter et al., 2022). As the pathogenesis is unclear and there is no definitive treatment available, it is of critical importance to identify the pathogenesis of IPF in order to find an effective therapeutic target (Spagnolo, Kropski et al., 2021). Based on the instant development of genomics and bioinformatics methods, exploring the alteration of specific genetic information and its regulatory mechanism during the development of IPF can not only gain a deep understanding the pathogenesis of IPF, but more importantly to provide possible biomarkers for early diagnosis and intervention of IPF.

Molecular biology studies had revealed that RNA N6-methyladenosine (m6A) modifications might take part in the development of respiratory diseases (Xiong, Hou et al., 2021). M6A is one of the commonly epigenetic modifications, which can regulate the expression level of certain genes after transcription through chemical modification without changing the mRNA sequence (Gui and Yuan 2021). Maladjustment of RNA methylation can lead to many diseases, including chronic obstructive pulmonary disease (COPD), respiratory tumors, IPF and pulmonary artery hypertension (Huang, Lv et al., 2020; Hu, Wang et al., 2021; Zhang, Huang et al., 2021).

EMT is a reprogramming process of epithelial to mesenchymal transition, in which epithelial cells lose intercellular adhesion and gain a greater ability to migrate and invade similar to mesenchymal cells (Phan, Paliogiannis et al., 2021). Lung epithelial cells can differentiate into myofibroblasts through EMT, which accelerates the fibrosis process (Moss, Ryter et al., 2022). A study demonstrated that methyltransferase-like 3 (METTL3) and m6A RNA modification were up-regulated in TGF-β-induced EMT of A549 and LC2/ad lung cancer cells (Wanna-Udom, Terashima et al., 2020). An animal experiment showed that m6A modification was activated in a mouse model of bleomycin-induced pulmonary fibrosis (Zhang, Huang et al., 2021). Recent research revealed that m6A inhibited the conversion of pri-miRNA-126 to mature miR-126, which in turn activated the downstream “PI3K-AKT-mTOR” signaling pathway, causing fibrosis in lung tissue (Han, Chu et al., 2020). Nonetheless, the roles of m6A modification in IPF are not fully demonstrated.

**FIGURE 1:**
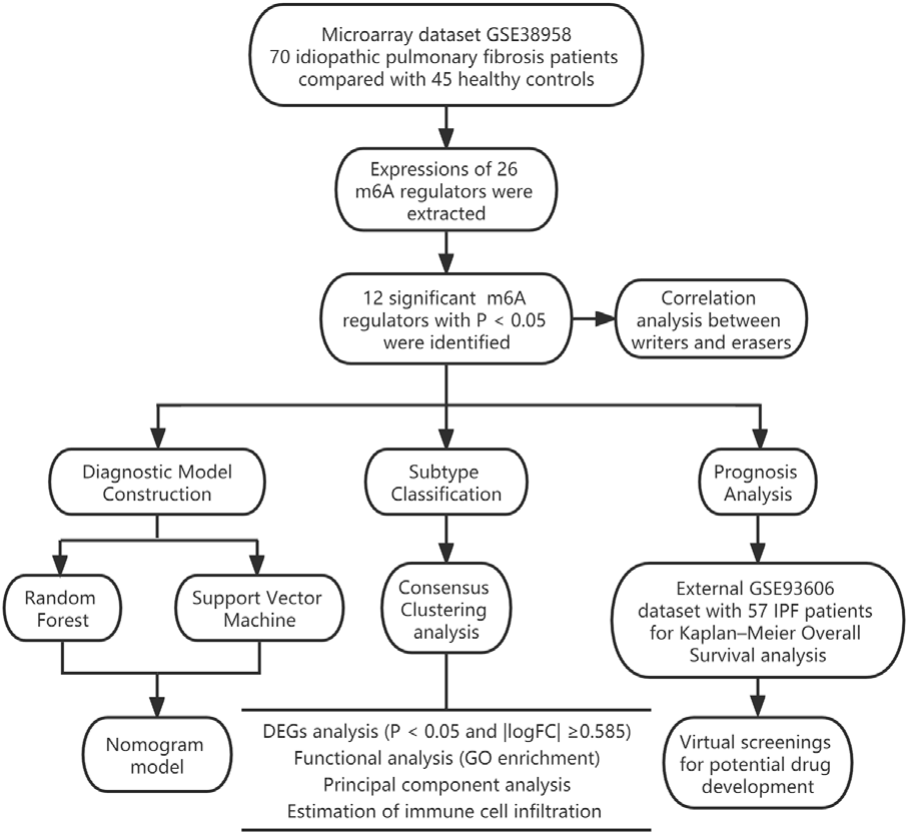
The study workflow.

In our research, we comprehensively assessed the effects of m6A regulators on the diagnosis and subtype categorization in IPF using the GSE38958 dataset. We constructed a gene model for prediction of IPF based on 11 candidate m6A regulators [leucine-rich PPR motif-containing protein (LRPPRC), METTL3, FTO alpha-ketoglutarate dependent dioxygenase (FTO), methyltransferase-like 14/16 (METTL14/METTL16), protein virilizer homolog (VIRMA), c-cbl-like 1 (CBLL1), fragile X messenger ribonucleoprotein 1 (FMR1), YTH domain containing 1/2 (YTHDC1/YTHDC2) and zinc finger CCCH-type containing 13 (ZC3H13)]. As a result, IPF patients could receive clinical benefits from this gene model. We also discovered two distinct m6A patterns, one of which was highly relevant to EMT, Th17 cell infiltration, Treg cell infiltration, innate immunity and Th1 immunity. This showed that m6A patterns could be applied to differentiate IPF and guide subsequent treatment. In addition, we identified two novel prognostic m6A regulators (LRPPRC and FTO) in IPF for the first time and obtained a total of 100 compounds or natural products as potential drugs targeting LRPPRC and FTO in the treatment of IPF with the aid of virtual screening technology. The workflow of this study is shown in Figure 1.

## Materials and methods

### Collection of IPF dataset

The GSE38958 dataset containing 45 healthy controls (HCs) and 70 IPF patients was downloaded from GEO database. The expressions of 26 m6A regulators were extracted from the dataset and further used to identify significant m6A regulators between HCs and IPF patients. The 26 m6A regulators were consisted of three parts, nine writers (METTL3/14//16, WTAP, VIRMA, ZC3H13, RBM15, RBM15B and CBLL1), fifteen readers (YTHDC1/2, YTHDF1/2/3, HNRNPC, FMR1, LRPPRC, HNRNPA2B1, IGFBP1/2/3, RBMX, ELAVL1 and IGF2BP1) and two erasers (FTO and ALKBH5) (Ilieva and Uchida 2022; Xie, Zhang et al., 2022).

### Optimal predicting model developed by random forest (RF) and support vector machine (SVM)

To develop a model to predict incidence of IPF, the RF and SVM model were respectively built as training models. Residual-related analysis and receiver operating characteristic (ROC) were utilized to assess the accuracy of models. RF is a regression tree technique using bootstrap aggregation and randomization of predictors to bring about a high degree of predictive accuracy based on the integration of traditional decision tree. SVM is also a supervised learning model, which is usually used for pattern recognition, classification (outlier detection) and regression analysis. In our study, we built a RF model to screen out candidate m6A regulators to predict the incidence of IPF using the “randomForest” package of R. The parameters were set as follow: ntrees = “500” and mtry = “3”. The X-axis represents the number of trees and Y-axis represents the error value of 10-fold cross validation. In this way, we selected the tree with a minimum error value as the optimal model for predicting the incidence of IPF. We also investigated the importance of the significant m6A regulators. In addition, a SVM model was built with the “kernlab” and “caret” packages of R. Each data point displayed in n-dimension spaces (“n” represents the amount of m6A regulators). An optimal hyperplane was then identified to perfectly distinguish IPF samples from HCs (Bao, Shi et al., 2020).

### Construction of a nomogram model

To further predict the prevalence of IPF, a nomogram model was built with the “rms” package of R based on the candidate m6A regulators. A calibration curve was then applied to determine whether the predictive value of the model was consistent with reality. Furthermore, we used decision curve analysis (DCA) and plotted a clinical impact curve to explore the benefit of decisions made by the model to IPF patients (Iasonos, Schrag et al., 2008).

### Exploration of distinct patterns based on the significant m6A regulators

Consensus clustering, as an unsupervised clustering method, selects a certain number of samples by resampling and specifies the number of clusters (k-means algorithm) to calculate the rationality under different cluster numbers. It is usually used to discover new disease subtypes, or to compare and analyze different disease subtypes. In our study, it was applied to explore different m6A patterns based on significant m6A regulators with the “ConsensusClusterPlus” package of R. (Wilkerson and Hayes 2010).

### Differentially expressed genes (DEGs) between different m6A patterns and functional analysis

DEGs analysis was conducted between different m6A patterns using the “limma” package. The screening criteria were set as follow: adjusted *p* < 0.05 and |logFC| ≥0.585. The filtered DEGs were then used for GO enrichment analysis to explore potential mechanisms involved in IPF using the “clusterProfiler” package. Lastly, the results were displayed in an enrichment circle diagram (Denny, Feuermann et al., 2018).

### Estimation of the m6A gene signature

Principal component analysis (PCA) was conducted to obtain the m6A scores of all the IPF patients to quantify the m6A patterns. Initially, PCA was employed to recognize distinct m6A patterns. M6A scores of each IPF patient were then calculated using the formula: m6A score = PC1_i,_ where PC1 refers to the principal component 1, and i refers to the DEGs expression (Zhang, Wu et al., 2020).

### Estimation of immune cell infiltration

We conducted single sample GSEA algorithm using the “GSVA”, “GSEABase”, and “limma” packages to calculate the immune cell infiltration of each IPF patient based on 23 immune cell gene sets. All members from the 23 immune cell gene sets were ranked according to their expression levels and then combined. As a result, the immune cell infiltration of each IPF patient was obtained (Zhang, Zhao et al., 2020).

### Survival analysis on significant m6A regulators

The external GSE93606 dataset containing 57 IPF patients was retrieved from GEO. We obtained clinical prognostic data and normalized gene expression data. We then carried out Kaplan–Meier overall survival (OS) analysis with the “survival” and “survminer” package of R to investigate the correlation between OS time and significant m6A regulators in IPF patients. The prognostic m6A regulators with a *p* < 0.05 were determined statistically significant.

### Virtual screening for potential drug

Virtual screening for potential drugs was carried out using the Vina protocol on Yinfo Cloud Computing Platform (https://cloud.yinfotek.com/). The crystal structures of two prognostic m6A regulators, FTO (PDB code: 3LFM, resolution: 2.50 Å) and LRPPRC (AF code: P42704-F1) were retrieved from RCSB Protein Data Bank (http://www.rcsb.org/) or AlphaFold Database (https://alphafold.ebi.ac.uk/). The Enamine HTS with about 175 million of compounds and the ZINC with about 130 thousand of natural products were used. Binding pocket was defined with the crystal ligand and the binding sites could be found in UniProt (https://www.uniprot.org/). If a protein did not have its binding crystal ligand and its binding sites could not be obtained from UniProt, the POCASA dataset (http://altair.sci.hokudai.ac.jp/g6/service/pocasa/) would be used to predict its binding pocket. The binding pocket predicted by POCASA with the maximum size was defined as the most potential pocket. Finally, binding modes of the top 25 docked compounds or natural products were visually analyzed and manual selection of hits was performed. AutoDock Vina software was utilized for semi-flexible docking (Trott and Olson 2010).

### Statistical analyses

We used R software (4.1.0 version) with corresponding packages mentioned above to carry out Linear regression analyses (LRA), Kruskal-Wallis tests (K-W tests) and Kaplan-Meier method (K-M method). LRA was utilized for investigation of the relationship between writers and erasers. K-W tests were utilized for the comparison of the differences between groups. K-M method was utilized for the overall survival analysis. In addition, two-tailed *p* < 0.05 was regarded statistically significant.

## Results

### Landscape of 26 m6A regulators in IPF

**FIGURE 2:**
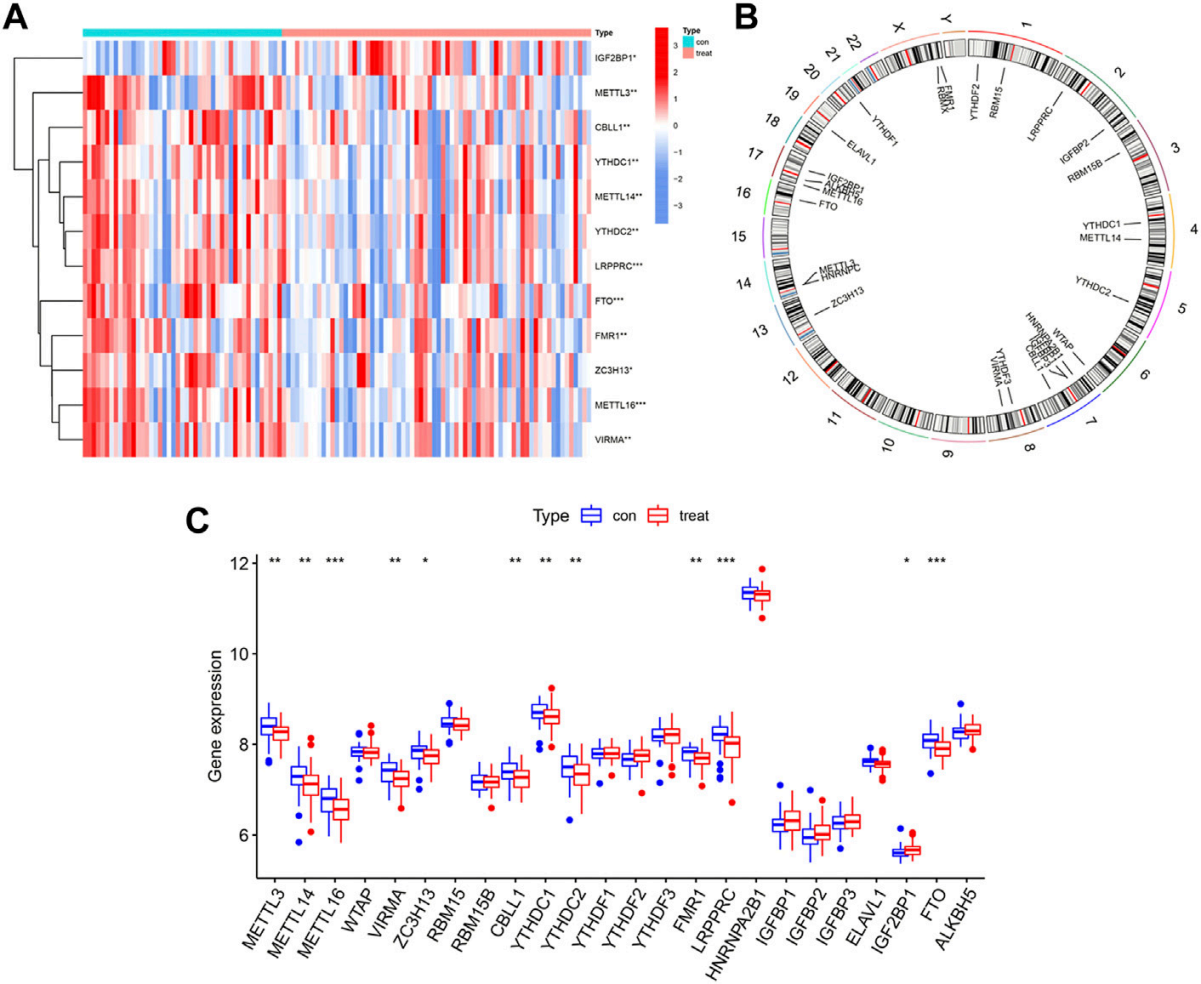
Landscape of m6A regulators in IPF. **(A)** Expression heatmap of 12 significant m6A regulators in healthy controls (Type: con) and IPF patients (Type: treat). **(B)** Chromosomal positions of the 26 m6A regulators. **(C)** Differential expression histogram of 12 significant m6A regulators identified between healthy controls (Type: con) and IPF patients (Type: treat). *p < 0.05, **p < 0.01, and ***p < 0.001.

We used the “limma” package of R to analyze differential expressions of 26 m6A regulators between HCs and IPF patients. In total, 24 m6A regulators with complete expression data were retrieved where twelve significant m6A regulators were identified and visualized in a heatmap and histogram. They were IGF2BP1, METTL3, CBLL1, YTHDC1, METTL14, YTHDC2, LRPPRC, FTO, FMR1, ZC3H13, METTL16, and VIRMA. In contrast with HCs, IGF2BP1 had been upregulated whereas the other 11 regulators had been downregulated in IPF patients (Figures 2A,C). Using the “RCircos” package, the 26 m6A regulators were mapped onto chromosomes (Figure 2B).

### Correlation of writers and erasers in IPF

**FIGURE 3:**
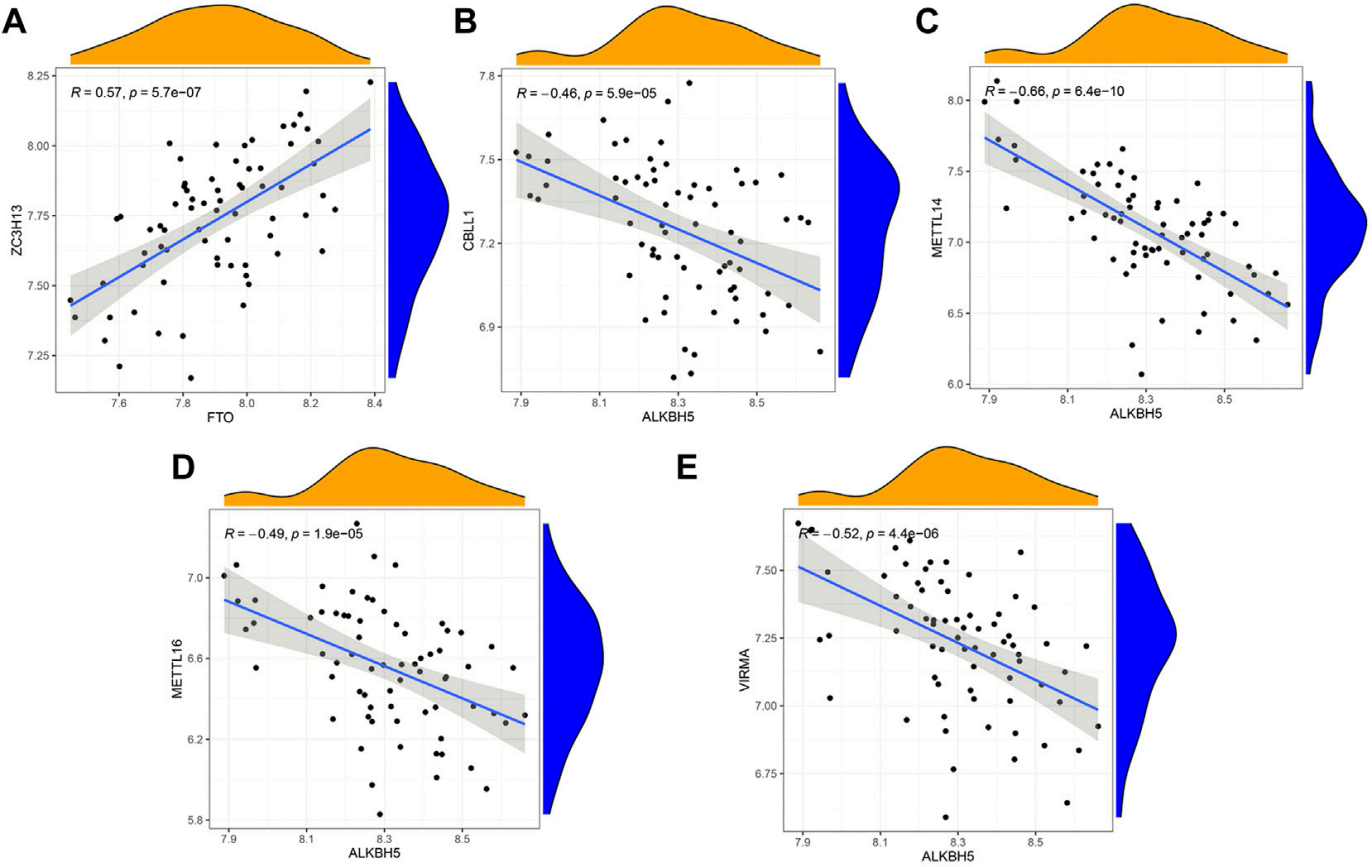
Correlation between writers and erasers in IPF **(A–E)** Writer genes: CBLL1, METTL14, METTL16, VIRMA and ZC3H13; eraser genes: ALKBH5 and FTO.

Based on linear regression analysis, we investigated the correlation between writers’ and erasers’ expression levels in IPF patients. A significant positive correlation between ZC3H13 and FTO was observed in IPF patients (Figure 3A). In addition, low expression levels of METTL14, METTL16, VIRMA, and CBLL1 were observed in IPF patients with high ALKBH5 expression levels (Figures 3B–E). Other writers did not show any correlation with erasers. The results here show correlation between the writers and erasers to varying degrees.

### Construction of the RF model and SVM model

**FIGURE 4:**
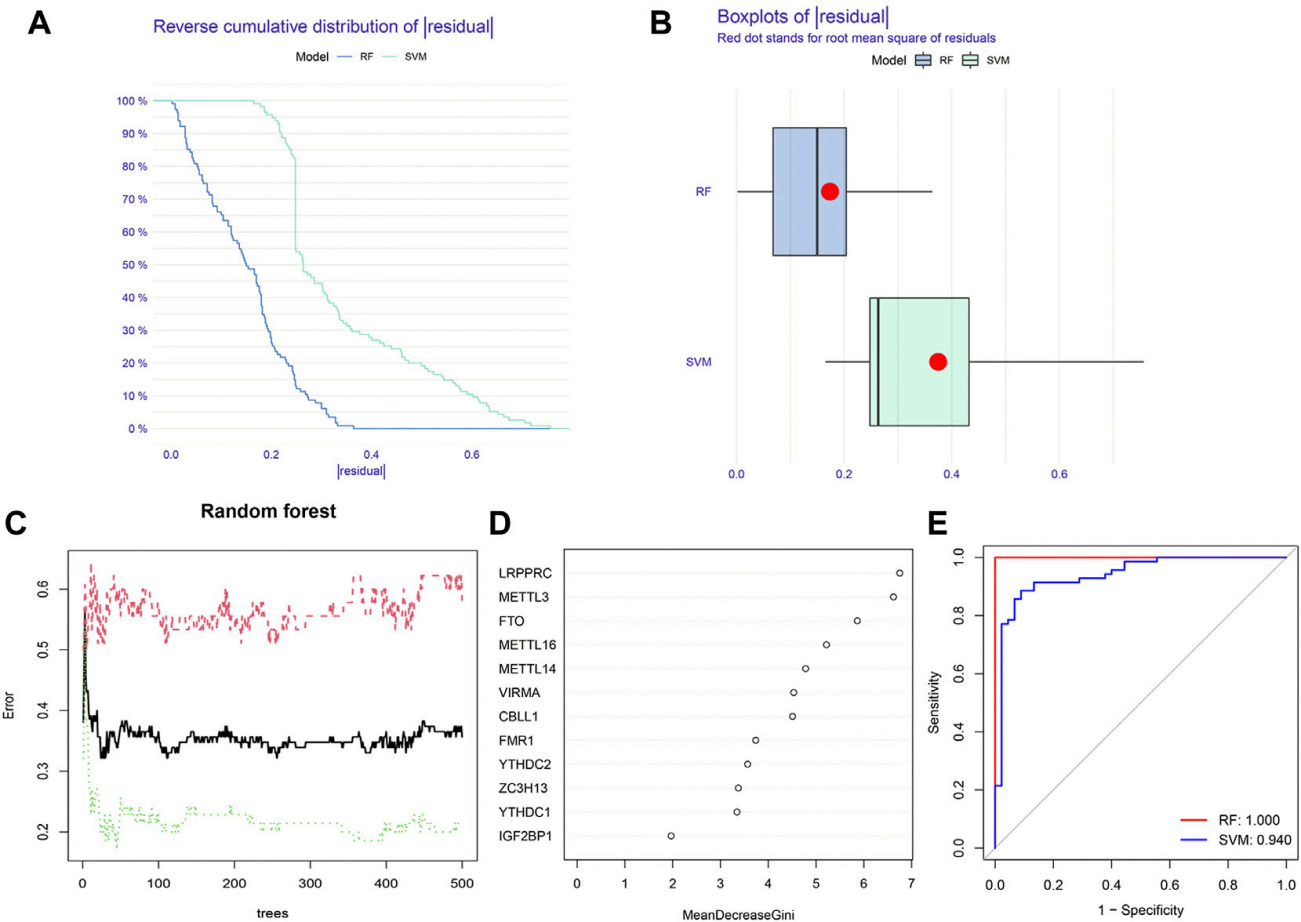
Construction of random forest (RF) model and support vector machine (SVM) model. **(A)** Reverse cumulative distribution of residual of RF and SVM model. **(B)** Boxplots of residual of RF and SVM model. **(C)** Cross-validation curve of RF model. **(D)** The importance of the 12 significant m6A regulators. **(E)** ROC curves of RF and SVM model.

Based on 12 significant m6A regulators, the RF model and SVM model were successively built to identify candidate m6A regulators in order to predict the incidence of IPF. According to the residual-related analysis (Figures 4A,B), the RF model showed minimal residuals, indicating that RF was a better method to predict the incidence of IPF. The RF trees-error curve showed that the RF model consisted of 11 m6A regulators has the lowest error rate (Figure 4C). Therefore, LRPPRC, METTL3, FTO, METTL16, METTL14, VIRMA, CBLL1, FMR1, YTHDC2, ZC3H13, and YTHDC1 were chosen as 11 candidate genes. After ranking each gene according to its importance, we visualized the significant m6A regulators (Figure 4D). Lastly, we plotted a ROC curve to measure the accuracy of the model. The result suggested again that the RF model was more accurate than SVM model (Figure 4E).

### Construction of the nomogram model

**FIGURE 5:**
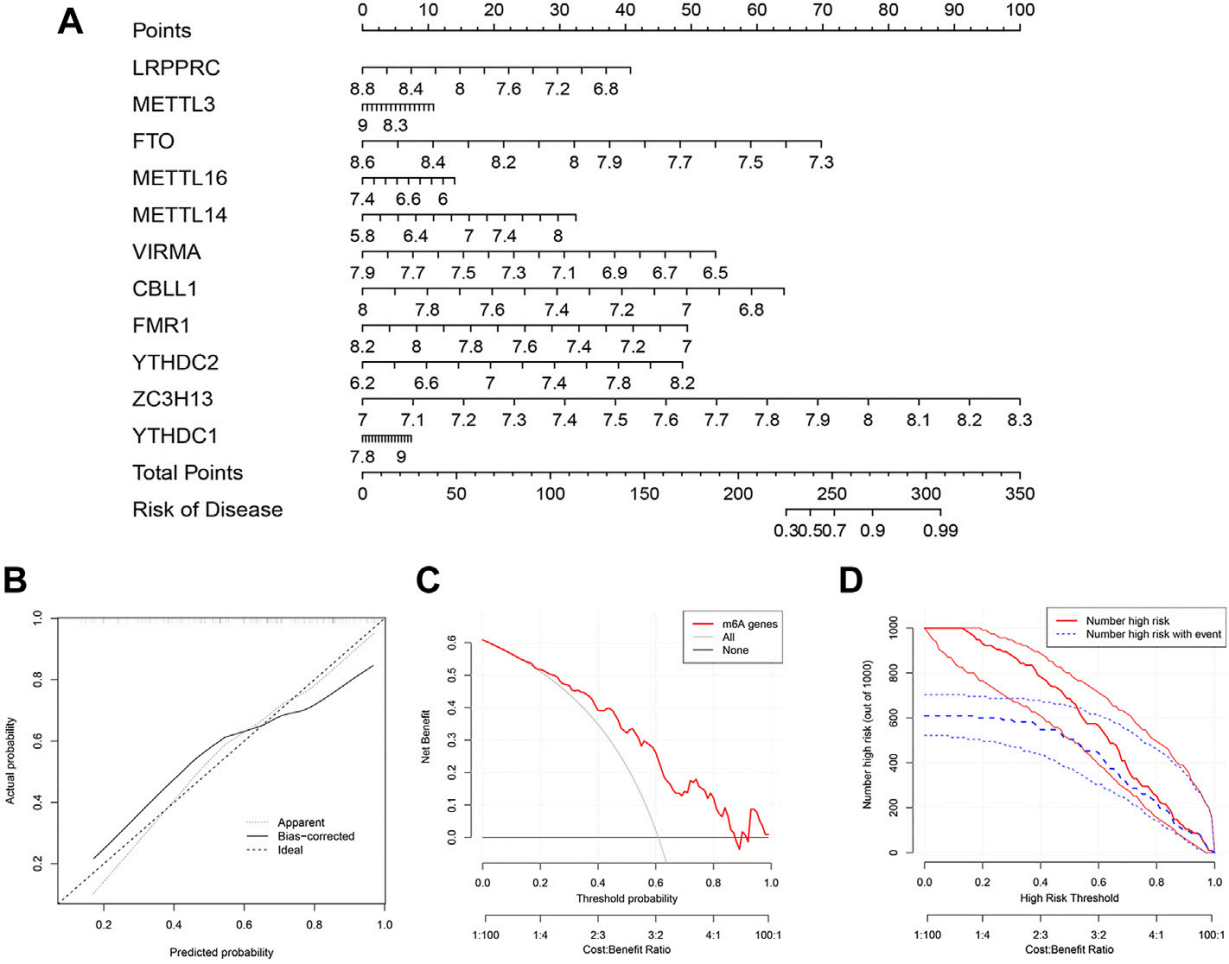
Construction of the nomogram model. **(A)** Nomogram model constructed by the 11 candidate m6A regulators. **(B)** Predictive value of nomogram model through a calibration curve. **(C)** Decisions curve analysis of nomogram model showing benefits to IPF patients. **(D)** Clinical impact curve of nomogram model.

To further predict the prevalence of IPF, a nomogram model was built using the “rms” package of R based on the 11 candidate m6A regulators (Figure 5A). The nomogram model was then verified for its accuracy by the calibration curves (Figure 5B). We found that decisions based on the nomogram might be beneficial to IPF patients according to the DCA curve. This was concluded based on the observation that the majority of the red line was higher than the black and grey lines ranging from zero to one (Figure 5C). As shown by the clinical impact curve, the model had an excellent predictive capacity (Figure 5D).

### Identification of two different m6A patterns by significant m6A regulators

**FIGURE 6:**
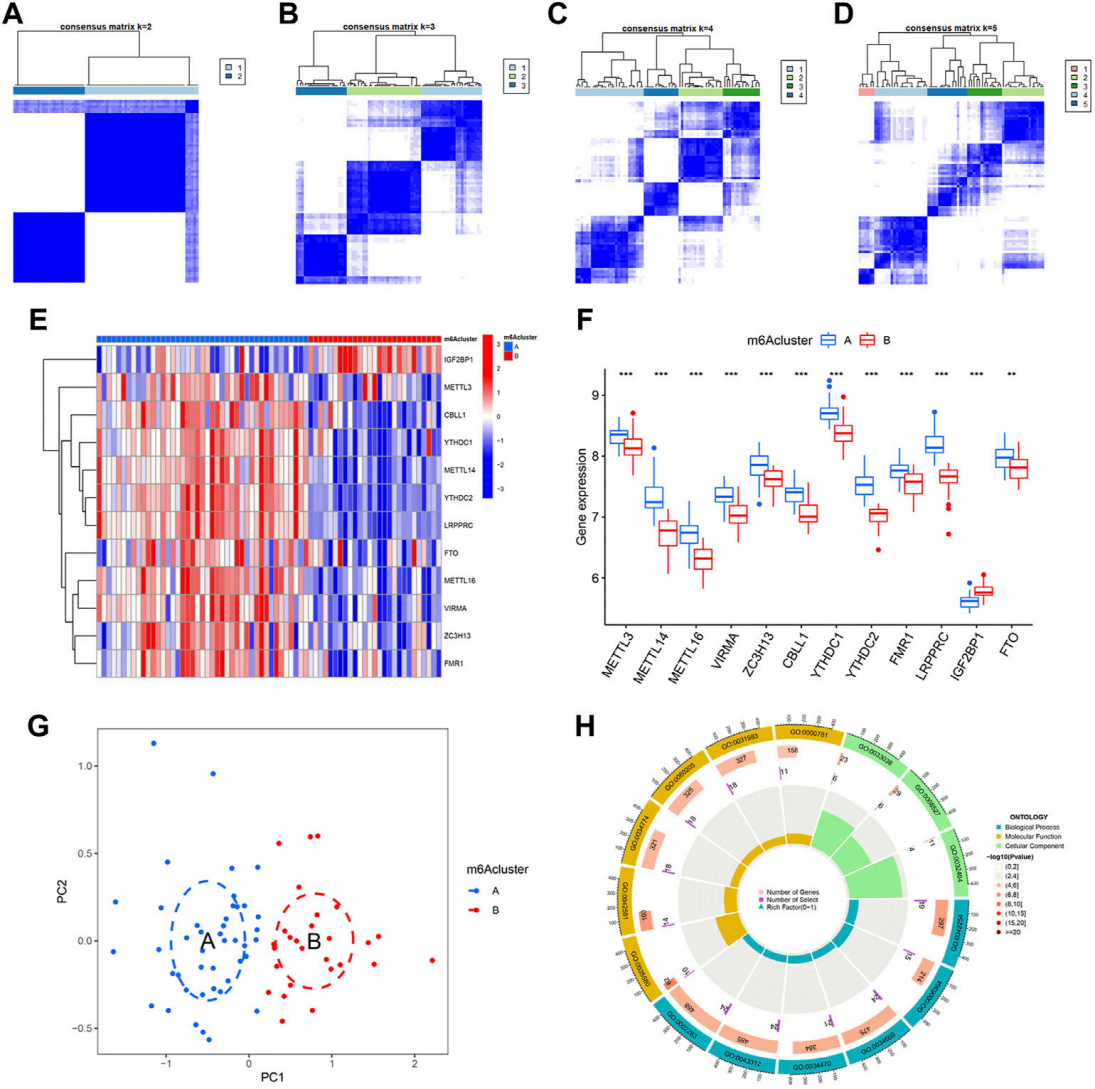
Consensus clustering of 12 significant m6A regulators in IPF. **(A–D)** Consensus matrices with cluster count from 2 to 5 showing an optimal cluster (clusterA and cluster B) with k = 2. **(E)** Expression heatmap in clusterA and clusterB. **(F)** Differential expression histogram in clusterA and clusterB. **(G)** Principal component analysis based on 12 significant m6A regulators showing a notable distinction between clusterA and clusterB. **(H)** Gene ontology enrichment showing potential biological functions of 402 m6A-related differentially expressed genes (DEGs) on the etiopathogenesis of IPF. *p < 0.05, **p < 0.01, and ***p < 0.001.

By using “ConsensusClusterPlus” package of R, we conducted the consensus clustering analysis for identification of different m6A patterns based on the 12 significant regulators. As shown in Figures 6A–D, 70 IPF patients were distinctly divided into two m6A patterns, clusterA (43 cases) and clusterB (27 cases). Heatmaps and histograms were used to demonstrate the differences in expression levels of the 12 significant m6A regulators. As seen in Figures 5E,F, when compare to clusterA, METTL3, METTL14, METTL16, VIRMA, ZC3H13, CBLL1, YTHDC1, YTHDC2, FMR1, LRPPRC, and FTO showed lower expression levels in clusterB while IGF2BP1 showed the opposite. According to the PCA, we could completely differentiate the two m6A patterns (Figure 5G). 402 m6A-related DEGs, with an adjusted *p* < 0.05 and a |logFC| ≥0.585, were then screened out from the two m6A patterns for further GO enrichment analysis. Detailed results of the 402 m6A-related DEGs can be found in Supplementary Table S1. As shown in Figure 6H, potential functions of the 402 DEGs were mainly involved in ncRNA metabolic process (GO:0034660), ncRNA processing (GO:0034470), neutrophil activation involved in immune response (GO:0002283), neutrophil degranulation (GO:0043312) and ribosome biogenesis (GO:0042254).

**FIGURE 7:**
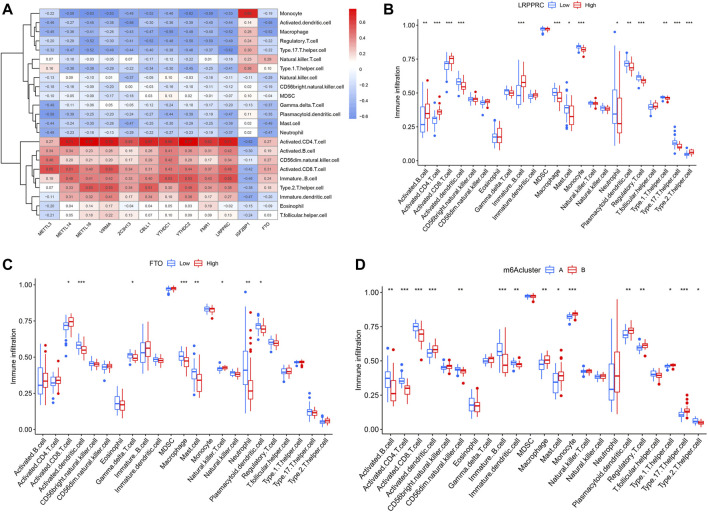
Single sample GSEA for immune infiltration. **(A)** Heatmap revealing relationship between immune cells and the 12 significant m6A regulators. **(B)** Distinction of immune cells infiltration between high and low LRPPRC expression subgroups. **(C)** Distinction of immune cells infiltration between high and low FTO expression subgroups. **(D)** Distinction of immune cells infiltration between clusterA and clusterB. *p < 0.05, **p < 0.01, and ***p < 0.001.

In addition, ssGSEA was conducted to obtain the number of immune cells in IPF patients and to explore the correlation of 12 significant m6A regulators with different immune cells. We observed that most of the m6A regulators, except for IGF2BP1, had positive correlations with activated CD4^+^ and CD8^+^ T cells, B cells, Th2 cells, and CD56^−^ natural killer cell, but negative correlations with Th17 cells, Treg cells, monocytes, dendritic cells, macrophages, mast cells and neutrophils (Figure 7A). We also found that IPF patients with higher LRPPRC and FTO expression levels had an enhanced adaptive immune cell infiltration and reduced innate immune cell infiltration in contrast to those with lower expression levels (Figures 7B,C). Lastly, the two m6A patterns were compared for their differential immune cell infiltration. The results demonstrated that clusterA had a linkage with adaptive immunity and Th2-dominant immunity while clusterB had a linkage with Th17 cell infiltration, Treg cell infiltration, innate immunity and Th1-dominant immunity (Figure 7D).

### Identification of two different m6A gene patterns and generation of the m6A gene signature

**FIGURE 8:**
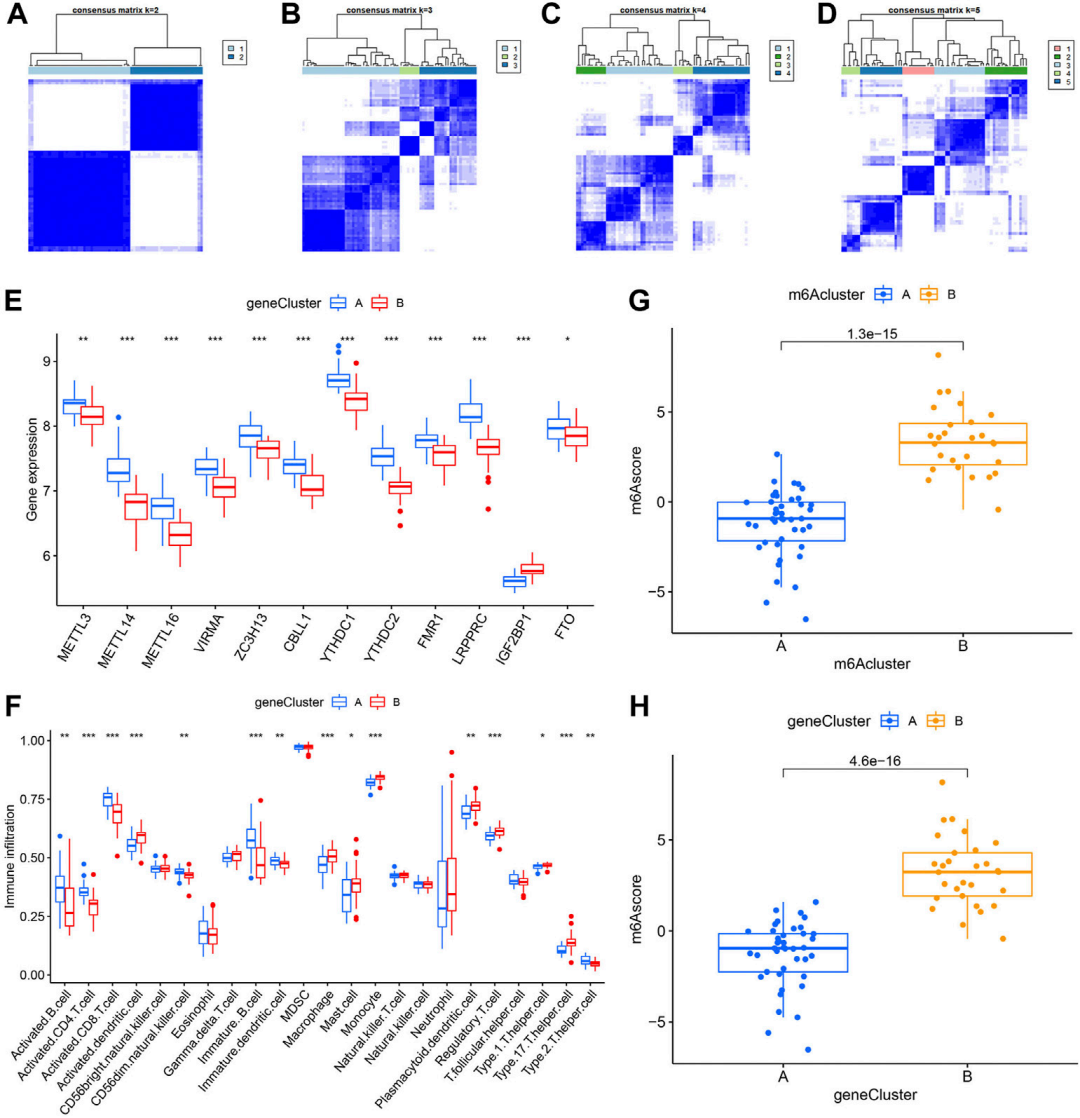
Consensus clustering of the 402 m6A-related DEGs in IPF. **(A–D)** Consensus matrices with cluster count from 2 to 5 showing an optimal cluster (gene clusterA and gene cluster B) with k = 2. **(E)** Differential expression histogram in gene clusterA and gene clusterB. **(F)** Distinction of immune cells infiltration between gene clusterA and gene clusterB. **(G)** Distinction of m6A score between clusterA and clusterB. **(H)** Distinction of m6A score between gene clusterA and gene clusterB. *p < 0.05, **p < 0.01, and ***p < 0.001.

**FIGURE 9:**
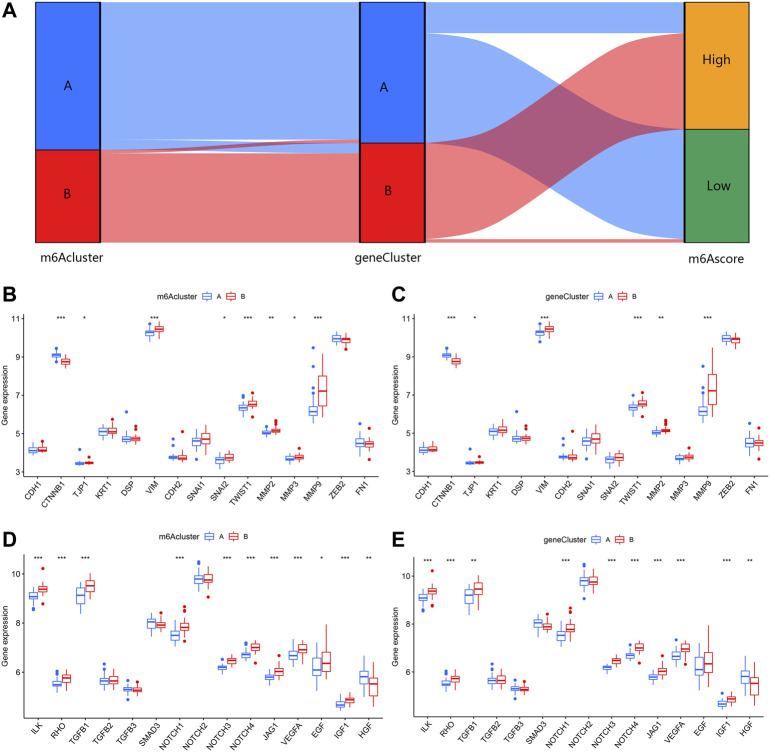
Effect of m6A patterns on differentiation of IPF. **(A)** Sankey diagram revealing relevance between m6A patterns, m6A gene patterns, and m6A scores. **(B)** Different expression level of epithelial markers and mesenchymal markers between clusterA and clusterB. **(C)** Different expression level of epithelial markers and mesenchymal markers between gene clusterA and gene clusterB. **(D)** Different expression level of regulatory factors of fibrosis between clusterA and clusterB. **(E)** Different expression level of regulatory factors of fibrosis between gene clusterA and gene clusterB. *p < 0.05, **p < 0.01, and ***p < 0.001.

In order to verify the m6A patterns, IPF patients were once again grouped into different genomic patterns using the consensus clustering analysis based on 402 m6A-related DEGs. Two different m6A gene patterns (gene clusterA and gene clusterB) were identified and found to be accordant with the grouping of m6A patterns (Figures 8A–D). Similar differentially expressed levels of the 12 significant m6A regulators and immune cells infiltration were observed in two different identified m6A gene patterns in contrast to two m6A patterns (Figures 8E,F). This also confirmed that grouping by consensus clustering algorithm was accurate. PCA algorithm was then used to obtain m6A scores for all IPF patients to quantify the m6A patterns. M6A scores of two different m6A patterns then compared, as well as the two different m6A gene patterns. As a result, clusterB or gene clusterB had higher m6A scores than clusterA or gene clusterA (Figures 8G,H). Sankey diagrams were used to visualize the relationship among m6A patterns, m6A gene patterns and m6A scores (Figure 9A).

### Role of m6A patterns in distinguishing IPF

For a further insight into roles of m6A patterns in IPF, we explored the correlations between m6A patterns and EMT-related gene set. The EMT-related gene set was consisted of epithelial markers, mesenchymal markers and regulatory factors of fibrosis, seen in Table 1. Epithelial markers were proven to be downregulated in fibrotic diseases, while mesenchymal markers and regulatory factors of fibrosis were confirmed to be positively correlated with the development of fibrosis. The results showed clusterB and gene clusterB had lower expressions of epithelial markers but higher expressions of mesenchymal markers (Figures 9B,C). Moreover, regulatory factors of fibrosis were highly expressed in clusterB and gene clusterB in contrast to those in clusterA and gene clusterA (Figures 9D,E). The above results indicated that both clusterB and gene clusterB were closely related to IPF featured with the EMT.

**TABLE 1:** The EMT-related gene set consisted of epithelial markers, mesenchymal markers and regulatory factors of fibrosis.

Type	Protein name	Gene symbol
Epithelial markers	E-cadherin/β-catenin/ZO-1/Cytokeratin/Desmoplakin	CDH1/CTNNB1/TJP1/KRT1/DSP
Mesenchymal markers	Vimentin/N-cadherin/Snail Family Transcriptional Repressor 1/Snail Family Transcriptional Repressor 2/Twist Family BHLH Transcription Factor 1/Twist Family BHLH Transcription Factor 2/Matrix Metallopeptidase 2/Matrix Metallopeptidase 3/Matrix Metallopeptidase 9/Zinc Finger E-Box Binding Homeobox 2/Fibronectin	VIM/CDH2/SNAI1/SNAI2/TWIST1/TWIST2/MMP2/MMP3/MMP9/ZEB2/FN1
Regulatory factors of fibrosis	Integrin Linked Kinase/Rhodopsin/Transforming Growth Factor Beta 1/Transforming Growth Factor Beta 2/Transforming Growth Factor Beta 3/SMAD Family Member 3/Notch Receptor 1/Notch Receptor 2/Notch Receptor 3/Notch Receptor 4/Jagged Canonical Notch Ligand 1/Vascular Endothelial Growth Factor A/Epidermal Growth Factor/Platelet Derived Growth Factor/Insulin Like Growth Factor 1/Hepatocyte Growth Factor/Fibroblast Growth Factor	ILK/RHO/TGFB1/TGFB2/TGFB3/SMAD3/NOTCH1/NOTCH2/NOTCH3/NOTCH4/JAG1/VEGFA/EGF/PDGF/IGF1/HGF/FGF

### Clinical prognostic value of significant m6A regulators in IPF

**FIGURE 10:**
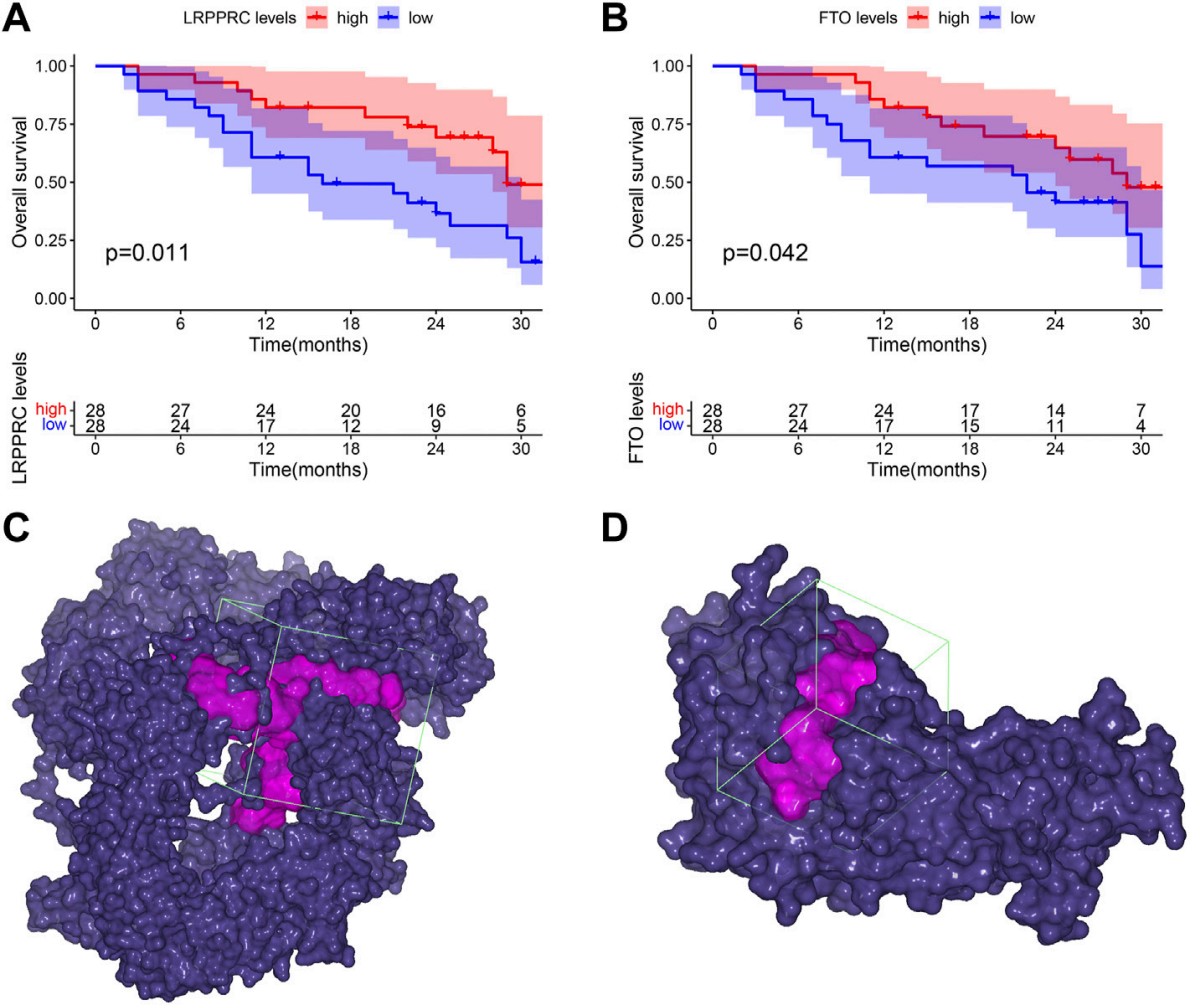
Clinical prognostic value of LRPPRC and FTO in IPF and their binding pockets for virtual screening. **(A)** Kaplan-Meier survival curve of the overall survival in high and low LRPPRC expressions subgroups. **(B)** Kaplan-Meier survival curve of the overall survival in high and low FTO expression subgroups. **(C)** Binding pocket of LRPPRC: core of pocket (−15.83, 4.206, 9.538) Å, size of pocket (40, 40, 40) Å. **(D)** Binding pocket of FTO: core of pocket (29.199, −7.339, −23.371) Å, size of pocket (26, 26, 26) Å.

To explore the clinical prognostic value of significant m6A regulators in IPF, we conducted OS analysis with an external GSE93606 dataset. The endpoint of OS was defined as death or a decline in FVC >10% over a 6-month period. We identified two OS-related genes, LRPPRC (*p* = 0.011) and FTO (*p* = 0.042), from 11 significant m6A regulators (Figures 10A,B). The higher expression levels of both LRPPRC and FTO were associated with a longer survival time or a better lung function in IPF patients.

### Virtual screening for potential drugs targeting LRPPRC and FTO

**FIGURE 11:**
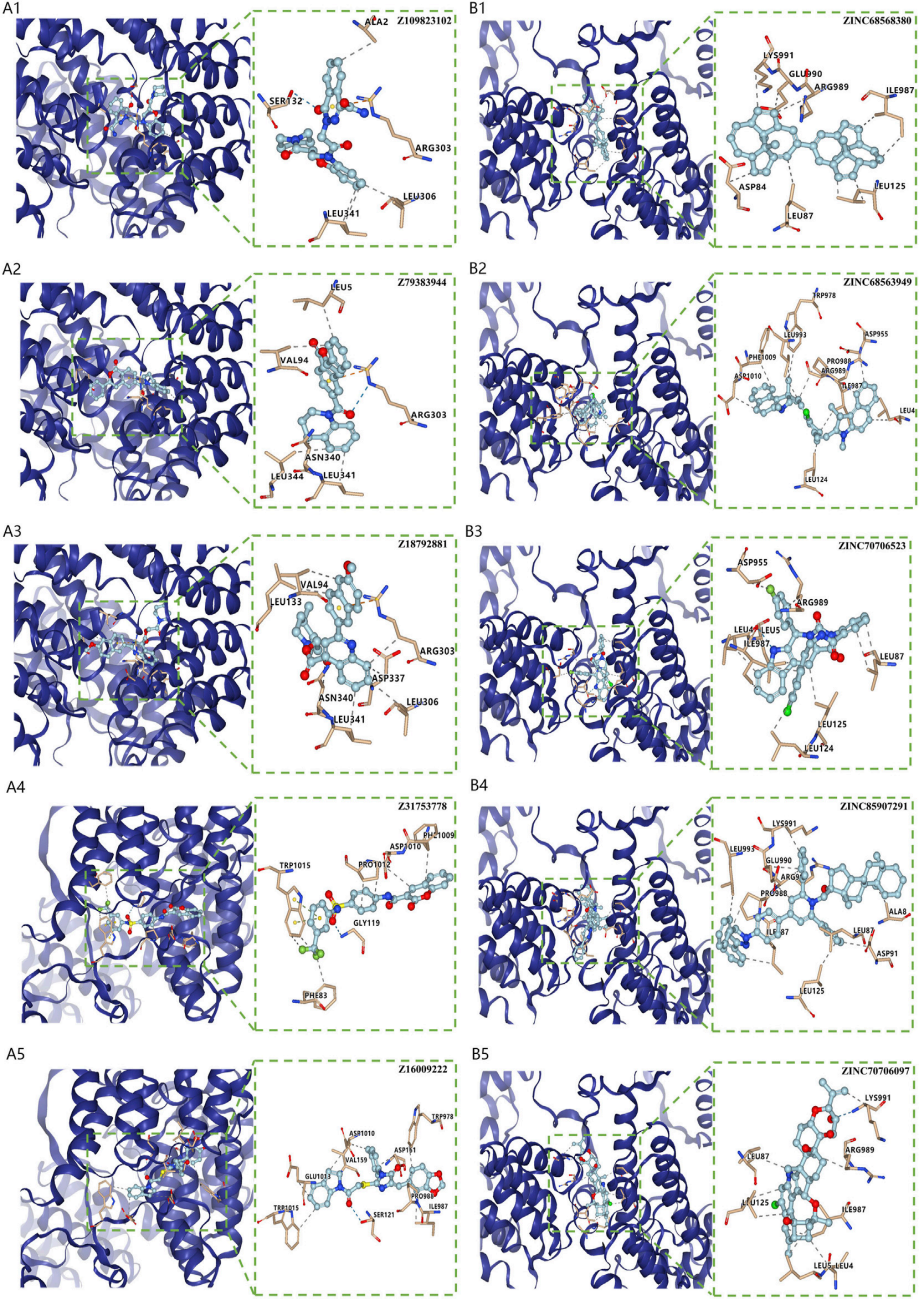
Virtual Screening for Potential Drugs Targeting LRPPRC. **(A)** The top five docked compounds (Z109823102, Z79383944, Z18792881, Z31753778, Z16009222) from Enamine HTS potentially targeting LRPPRC. Interaction type between each compound and LRPPRC: Z109823102 (hydrophobic interaction, hydrogen bond, π-cation interaction), Z79383944 (hydrophobic interaction, hydrogen bond, π-cation interaction), Z18792881 (hydrophobic interaction,π-cation interaction), Z31753778 (hydrophobic interaction, hydrogen bond, π-π stacking), Z16009222 (hydrophobic interaction, hydrogen bond). **(B)** The top five docked natural products (ZINC68568380, ZINC68563949, ZINC70706523, ZINC85907291, ZINC70706097) from ZINC potentially targeting LRPPRC. Interaction type between each product and LRPPRC: ZINC68568380 (hydrophobic interaction), ZINC68563949 (hydrophobic interaction), ZINC70706523 (hydrophobic interaction, hydrogen bond), ZINC85907291 (hydrophobic interaction), ZINC70706097 (hydrophobic interaction, hydrogen bond).

**FIGURE 12:**
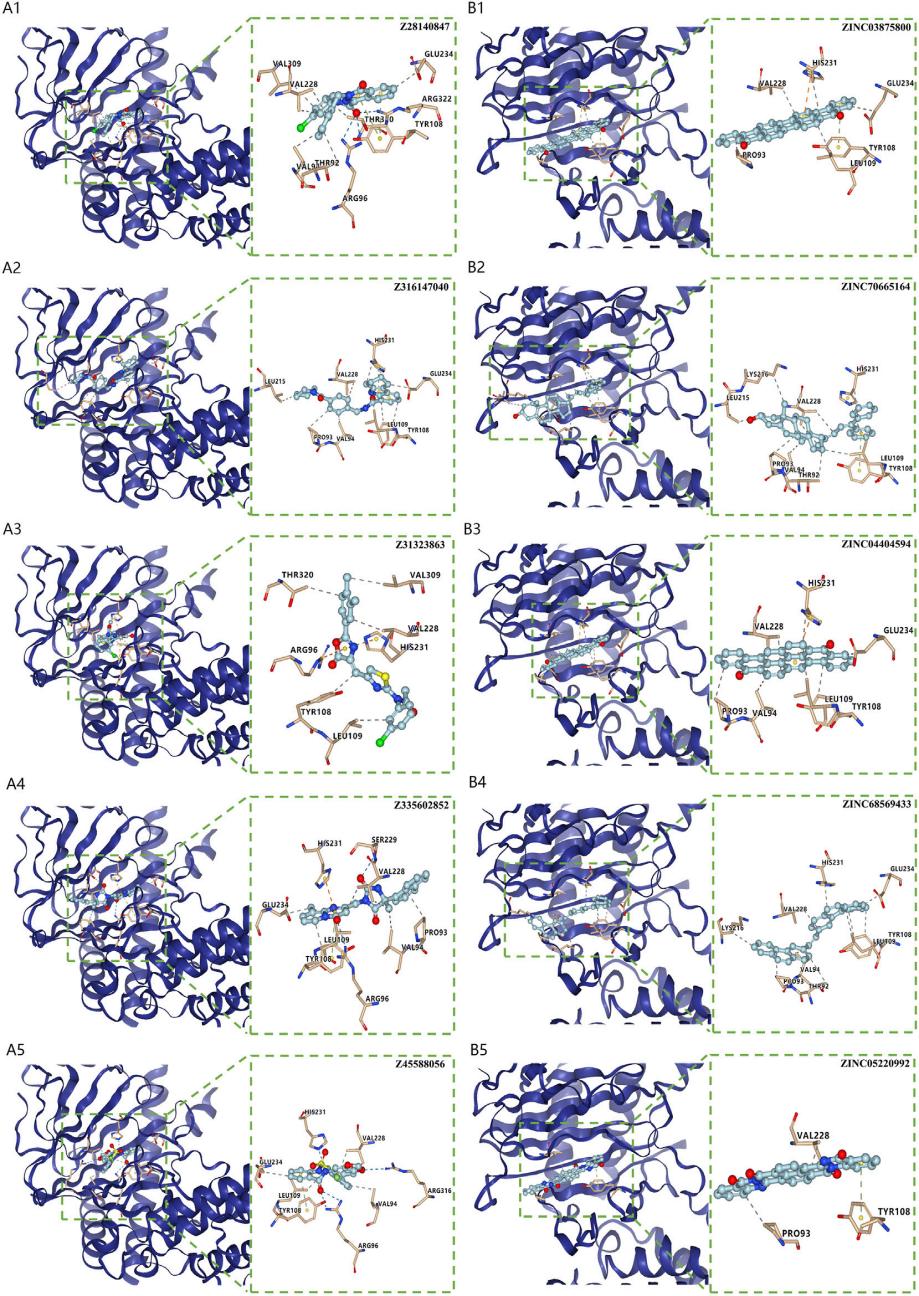
Virtual Screening for Potential Drugs Targeting FTO. **(A)** The top five docked compounds (Z28140847, Z316147040, Z31323863, Z335602852, Z45588056) from Enamine HTS potentially targeting FTO. Interaction type between each compound and FTO: Z28140847 (hydrophobic interaction, hydrogen bond, π-π stacking), Z316147040 (hydrophobic interaction, π-π stacking), Z31323863 (hydrophobic interaction, π-cation interaction), Z335602852 (hydrophobic interaction, π-π stacking), Z31323863 (hydrophobic interaction, π-cation interaction, π-π stacking), Z45588056 (hydrophobic interaction, hydrogen bond, π-π stacking). **(B)** The top five docked natural products (ZINC03875800, ZINC70665164, ZINC04404594, ZINC68569433, ZINC05220992) from ZINC potentially targeting FTO. Interaction type between each product and FTO: ZINC03875800 (hydrophobic interaction, π-cation interaction, π-π stacking), ZINC70665164 (hydrophobic interaction, π-π stacking), ZINC04404594 (hydrophobic interaction, π-cation interaction), ZINC68569433 (hydrophobic interaction), ZINC05220992 (hydrophobic interaction, π-π stacking).

Vina protocol on Yinfo Cloud Computing Platform was used to conduct virtual screening for potential drugs targeting LRPPRC and FTO. Binding pocket of LRPPRC was predicted by POCASA and shown in Figure 10C. Binding pocket of FTO was defined with crystal ligand (Han, Niu et al., 2010; Huang, Yan et al., 2015; Wang, Hong et al., 2015), shown in Figure 10D. We chose the top 25 docked compounds with an affinity lower than −9.6 kcal/mol (the top five: Z109823102, Z79383944, Z18792881, Z31753778, Z16009222) from Enamine HTS and top 25 docked natural products with an affinity lower than −10.2 kcal/mol (the top five: ZINC68568380, ZINC68563949, ZINC70706523, ZINC85907291, ZINC70706097) from ZINC as potential drugs for targeting LRPPRC (Figures 11A,B). Similarly, we chose the top 25 docked compounds with an affinity lower than −9.3 kcal/mol (the top five: Z28140847, Z316147040, Z31323863, Z335602852, Z45588056) from Enamine HTS and top 25 docked natural products with an affinity lower than −10.6 kcal/mol (ZINC03875800, ZINC70665164, ZINC04404594, ZINC68569433, ZINC05220992) from ZINC as potential drugs for targeting FTO (Figures 12A,B). All the virtual screening scores and the detailed information and structure of 100 potential drugs targeting LRPPRC and FTO were attached as Supplementary Tables S2–S5.

## Discussion

IPF is a chronic inflammatory interstitial lung disease of unknown origin, characterized by diffuse alveolitis and disruption of alveolar structure, leading to diffuse interstitial lung fibrosis (Richeldi, Collard et al., 2017; Phan, Paliogiannis et al., 2021). Molecular biology studies have revealed that aberrant m6A modifications exert influence on the progression of many diseases and may be critical to respiratory diseases (Jiang, Liu et al., 2021; Xiong, Hou et al., 2021). Nonetheless, the role of m6A regulators in IPF has not been fully understood. In our study, we aimed to explore roles of m6A regulators in IPF and to investigate potential therapeutic targets on this basis.

Firstly, 12 significant m6A regulators were screened out through DEGs analysis between HCs and IPF patients. An RF model was then constructed to choose 11 candidate m6A regulators (LRPPRC, METTL3, FTO, METTL16, METTL14, VIRMA, CBLL1, FMR1, YTHDC2, ZC3H13, and YTHDC1) to predict the incidence of IPF. On this basis, a nomogram model was built, and the DCA curve suggested that decisions according to the model may be beneficial to IPF patients. IGF2BP1, a subtype of the IGF2BPs family, serves as a m6A reader that recognizes GG (m6A)C sequence of thousands of mRNA, thus enhances its targeting mRNAs’ stability and translation in an m6A-depedent way (Huang, Weng et al., 2018). CBLL1 is a key part of the m6A methyltransferase complex that mediates m6A methylation of RNAs (Růžička, Zhang et al., 2017; Yue, Liu et al., 2018). The m6A reader YTHDC1 modulates mRNA splicing by regulation and chemotaxis of pre-mRNA splicing factors so that it can reach the binding domains of its target mRNAs (Xiao, Adhikari et al., 2016). METTL14, a critical part of methyltransferase complex, functions together with METTL3, thus combined into a stabile structure that plays an integral role in m6A deposition and enhances catalysis (Liu, Yue et al., 2014). YTHDC2, one of YTH proteins, selectively fuses with the motif of m6A, thereby increasing the translation effect of its targets and reducing mRNA abundance (Hsu, Zhu et al., 2017). LRPPRC conducts as an RNA-binding protein that regulates mRNAs encoded by mitochondria DNA and transactivates nuclear DNAs(Zhang, Gu et al., 2020). Furthermore, LRPPRC regulates various biological functions, such as energy metabolism and maturation (Cui, Wang et al., 2019). FTO localizes in nuclear speckles and serves as an erase to remove the m6A modifications in RNA and to regulate mRNA splicing (Jia, Fu et al., 2011; Zhao, Yang et al., 2014). FMR1, an RNA binding protein highly expressed in brain neurons, regulates the transcription and translation of synapse-related genes by modulating stability of its m6A-marked mRNAs (Zhang, Kang et al., 2018). ZC3H13, one of the zinc finger proteins, anchors WTAP, VIRMA and CBLL1 in the caryon to enhance m6A methylation (Wen, Lv et al., 2018). As part of the methyltransferase complex, METTL16 catalyzes m6A modification and acts as a methyltransferase of U6 spliceosomal small nuclear RNA (Pendleton, Chen et al., 2017). VIRMA is another essential part of the intact m6A methyltransferase complex, which not only preferentially regulates m6A methylation in 3′UTR and near stop codon, but also correlates with polyadenylation (Yue, Liu et al., 2018). These 11 candidate m6A regulators have been demonstrated a linkage with tumorigenesis and progression, involving hyperplasia, metastasis, synchronous radiotherapy resistance, metabolic reprogramming and prognosis (Zhu, Zhou et al., 2019; He, Li et al., 2020; Gao, Ye et al., 2021; An and Duan 2022). Moreover, they have been proven to participate in a variety of other diseases, such as psychiatric disorders, Alzheimer’s disease, metabolic syndrome and cardiovascular diseases, and may exert influence on COPD and IPF (Huang, Lv et al., 2020; Jiang, Liu et al., 2021; Zhang, Huang et al., 2021). However, relationship between the 11 candidate m6A regulators and IPF has not been fully studied. We hope that our research can reveal how m6A modification and m6A regulators contribute to the pathogenesis of IPF, in order to provide new ideas for future research on m6A regulators in IPF.

Nowadays, experts commonly consider EMT as one of the crucial etiopathogenesis of IPF. EMT is a biological process characterized by a loss of epithelial markers (E-Cadherin, etc.), but accumulation of mesenchymal markers (N-Cadherin, Vimentin, Fibronectin, etc.) as well as activation of regulatory markers of fibrosis (Snail, Slug, Twist, etc.), leading to a transition from epithelial features to mesenchymal phenotype (Phan, Paliogiannis et al., 2021; Moss, Ryter et al., 2022). When lung is damaged by various pathogens, numerous immune cells and various immunity related signals are activated, resulting in inflammatory conditions and aggravating EMT. Activation of innate immunity, including monocyte, macrophage, dendritic cell and mast cell, is responsible for progression and a poor prognosis of IPF(Scott, Quinn et al., 2019; Overed-Sayer, Miranda et al., 2020; Bocchino, Zanotta et al., 2021; Mattoo and Pillai 2021; Shenderov, Collins et al., 2021; van Geffen, Deißler et al., 2021). Th1/Th2 imbalance also contributes to aggravation of IPF, but which of them plays a dominant role remains controversial (Desai, Winkler et al., 2018; Planté-Bordeneuve, Pilette et al., 2021; Shenderov, Collins et al., 2021). Th17 cell and Treg cell have been proven positively associated with the development of IPF (Shenderov, Collins et al., 2021; van Geffen, Deißler et al., 2021). Moreover, if the inflammation is sustained, EMT is enhanced and prolonged through increased fibroblast proliferation. Eventually fibrous tissue progressively takes the place of functionally normal tissue, leading to progressive structural and functional dysfunction of lung parenchyma (Phan, Paliogiannis et al., 2021). In our study, consensus clustering algorithm was utilized to identify two m6A patterns (clusterA and clusterB) based on 12 significant m6A regulators. ClusterB was highly correlated to Th17 cell infiltration, Treg cell infiltration, innate immunity and Th1-dominant immunity and lower-level expression of epithelial markers but higher-level expression of mesenchymal markers and regulatory markers of fibrosis, suggesting that clusterB is highly related to IPF. Besides, we verified the reliability of these results in m6A gene patterns based on the 402 m6A-related DEGs. We also calculated the m6A score of all IPF patients to quantify the m6A patterns and found clusterB or gene cluster had a higher m6A score than clusterA or gene clusterA.

Furthermore, we firstly discovered two prognostic m6A regulators (LRPPRC and FTO) from 12 significant m6A regulators using an external IPF dataset. In our study, both of them were down-regulated in IPF patients compared with HCs. The higher expression levels of LRPPRC (*p* = 0.011) and FTO (*p* = 0.042) were significantly linked to a longer survival time or a better lung function in IPF patients. We also found that the lower expression levels of LRPPRC and FTO were highly relevant to an enhance innate immune cell infiltration, which was generally considered as a critical cause of incidence or aggravation for IPF. These results fully confirmed that both LRPPRC and FTO could serve as protective factors as well as potential therapeutic targets for IPF patients. FTO has already been confirmed to be a protective m6A regulator in myocardial fibrosis (Mathiyalagan, Adamiak et al. 2019, Ju, Liu et al., 2021), while the function of LRPPRC on fibrotic diseases is still unknown. It is supposed that the dysregulation or dysfunction of LRPPRC may play critical roles in diseases caused by PI3K/AKT/mTOR and JAK/STAT pathway dysregulation (Cui, Wang et al., 2019), including IPF (Woodcock, Eley et al., 2019; Montero, Milara et al., 2021; Wasnick, Korfei et al., 2022). But such supposition requires further study. Moreover, the top 25 docked compounds from Enamine HTS and top 25 natural products from ZINC were selected by virtual screening as potential drugs in the treatment of IPF, targeting LRPPRC and FTO. Most of them had various kinds of interaction with LRPPRC and FTO, such as hydrogen bond, π-π stacking and hydrophobic interaction, indicating a good bonding property (O'Connor and Cummins 2017; Shen, Xia et al., 2017; Zhuang, Wang et al., 2019). However, further verification is needed for these potential drugs before they can be used in practice Shen et al. (2022).

## Conclusion

M6A regulators exert a crucial effect on the onset and progression of IPF. Our study filtered out 11 candidate m6A regulators to accurately predict the prevalence of IPF using a nomogram model. Furthermore, we developed two different m6A patterns, one of which (clusterB) showed a close link to IPF. Interestingly and more importantly, we discovered two novel OS-related m6A regulators (LRPPRC and FTO) that could predict the prognosis of IPF patients. They may serve as protective factors and therapeutic targets. Our investigation of m6A patterns may provide clues for clinical diagnosis, prognosis and targeted therapeutic drugs development for IPF in the future.

## Data Availability

The datasets presented in this study can be found in online repositories. The names of the repository/repositories and accession number(s) can be found in the article/Supplementary Material.
