# Corrigendum: Effect of M6A regulators on diagnosis, subtype classification, prognosis and novel therapeutic target development of idiopathic pulmonary fibrosis

**DOI:** 10.3389/fphar.2022.1117317

**Published:** 2022-12-16

**Authors:** Guirui Huang, Shuaiyang Huang, Hongsheng Cui

**Affiliations:** Department of Respiratory, The Third Affiliated Hospital, Beijing University of Chinese Medicine, Beijing, China

**Keywords:** idiopathic pulmonary fibrosis, m6A regulators, diagnostic model, consensus clustering, prognostic markers, novel therapeutic targets

In the published article, there was an error. The dataset from GEO database we used for analysis was GSE38958 but written or revised wrongly in section of **Abstract** and **Introduction**.

A correction has been made to **Abstract**, “Paragraph 1.” This sentence previously stated:

“In this study, 12 significant m6A regulators were filtered out between healthy controls and IPF patients using GSE33566 dataset.”

The corrected sentence appears below:

“In this study, 12 significant m6A regulators were filtered out between healthy controls and IPF patients using GSE38958 dataset.”

Another correction has been made to **Introduction**, “Paragraph 4.” This sentence previously stated:

“In our research, we comprehensively assessed the effects of m6A regulators on the diagnosis and subtype categorization in IPF using the GSE33566 dataset.”

The corrected sentence appears below:

“In our research, we comprehensively assessed the effects of m6A regulators on the diagnosis and subtype categorization in IPF using the GSE38958 dataset.”

The authors apologize for this error and state that this does not change the scientific conclusions of the article in any way. The original article has been updated.

