# Supplementary material for: Effect of M6A regulators on diagnosis, subtype classification, prognosis and novel therapeutic target development of idiopathic pulmonary fibrosis
Source: Front Pharmacol. 2022 Nov 28;13:993567. doi: 10.3389/fphar.2022.993567 (PMC9742476; doi:10.3389/fphar.2022.993567)
Supplement: Supplementary file 1 [file DataSheet1.docx]

Supplementary Material

Effect of M6A Regulators on Diagnosis, Subtype Classification, Prognosis and New Therapy Target Development of Idiopathic Pulmonary Fibrosis

Guirui Huang, Shuaiyang Huang, Hongsheng Cui

# Supplementary Tables

**Table S1 402 m6A-related DEGs with a P < 0.01 and a |logFC| ≥0.585 between CluseterB and ClusterA**

| **NO.** | **Gene Symbol** | **logFC** | **AveExpr** | **t** | ***P*.Value** | **adj.*P*.Val** | **B** |
| --- | --- | --- | --- | --- | --- | --- | --- |
| 1 | TTC37 | -0.732541942 | 7.498075643 | -11.04356919 | 3.94E-17 | 4.35E-13 | 28.58235273 |
| 2 | ATR | -0.672898504 | 6.859057271 | -10.8837896 | 7.62E-17 | 4.35E-13 | 27.93966212 |
| 3 | TMEM168 | -0.601370897 | 7.138144329 | -10.75806317 | 1.28E-16 | 4.35E-13 | 27.43204657 |
| 4 | FASTKD2 | -0.732118948 | 5.9431551 | -10.74995293 | 1.33E-16 | 4.35E-13 | 27.39924557 |
| 5 | TTC21B | -0.672333407 | 6.250402214 | -10.67544832 | 1.81E-16 | 4.35E-13 | 27.09760569 |
| 6 | ZNHIT6 | -0.901374717 | 6.706818686 | -10.67517128 | 1.81E-16 | 4.35E-13 | 27.09648303 |
| 7 | LRPPRC | -0.589706955 | 7.965962929 | -10.6475831 | 2.03E-16 | 4.35E-13 | 26.98464619 |
| 8 | WDR75 | -0.729425421 | 7.217891171 | -10.46779818 | 4.30E-16 | 6.14E-13 | 26.25399418 |
| 9 | GTPBP10 | -0.888983807 | 6.332101571 | -10.4480927 | 4.67E-16 | 6.16E-13 | 26.17372016 |
| 10 | PTCD3 | -0.603198315 | 7.447351886 | -10.37053375 | 6.46E-16 | 7.91E-13 | 25.85741331 |
| 11 | SMARCAD1 | -0.802763905 | 6.678160029 | -10.35373844 | 6.93E-16 | 7.92E-13 | 25.78884348 |
| 12 | NUP133 | -0.585014068 | 6.377709414 | -10.28896699 | 9.09E-16 | 9.17E-13 | 25.52416004 |
| 13 | WDR36 | -0.590115114 | 7.278651729 | -10.12535338 | 1.81E-15 | 1.19E-12 | 24.85389541 |
| 14 | TRMT13 | -0.726031502 | 6.926647743 | -10.04370875 | 2.55E-15 | 1.51E-12 | 24.51856361 |
| 15 | ZSCAN26 | -0.605004262 | 6.065005771 | -9.974467282 | 3.41E-15 | 1.85E-12 | 24.23374074 |
| 16 | UCHL5 | -0.7002378 | 5.846184114 | -9.972243752 | 3.44E-15 | 1.85E-12 | 24.22458785 |
| 17 | CASD1 | -0.76778104 | 6.845116629 | -9.971471717 | 3.46E-15 | 1.85E-12 | 24.22140977 |
| 18 | MIER3 | -0.669887549 | 6.438526314 | -9.914515226 | 4.40E-15 | 2.21E-12 | 23.98681717 |
| 19 | TMEM209 | -0.621155377 | 7.527537986 | -9.895732297 | 4.76E-15 | 2.27E-12 | 23.90939778 |
| 20 | UTP15 | -0.806125858 | 5.406889043 | -9.884699799 | 4.99E-15 | 2.27E-12 | 23.86391126 |
| 21 | RIOK2 | -0.782438118 | 6.058811929 | -9.882900824 | 5.02E-15 | 2.27E-12 | 23.85649327 |
| 22 | SLC4A7 | -0.768891637 | 7.028445 | -9.86980187 | 5.31E-15 | 2.33E-12 | 23.80247276 |
| 23 | ZBTB41 | -0.662978577 | 6.525246343 | -9.668803565 | 1.24E-14 | 4.27E-12 | 22.97194025 |
| 24 | ZNF480 | -0.766611766 | 5.501514286 | -9.660258919 | 1.29E-14 | 4.34E-12 | 22.93656891 |
| 25 | DHX36 | -0.66501946 | 7.147239843 | -9.608883515 | 1.60E-14 | 5.00E-12 | 22.72378983 |
| 26 | TRMT11 | -0.75279592 | 6.428559557 | -9.586662642 | 1.76E-14 | 5.12E-12 | 22.63170327 |
| 27 | TTC14 | -0.671760363 | 7.911746943 | -9.578777732 | 1.82E-14 | 5.12E-12 | 22.59901911 |
| 28 | SLC38A9 | -0.589935476 | 6.793663343 | -9.569757201 | 1.89E-14 | 5.15E-12 | 22.5616226 |
| 29 | GLMN | -0.805814873 | 5.663357157 | -9.559214453 | 1.98E-14 | 5.31E-12 | 22.51790862 |
| 30 | TOMM70 | -0.615902094 | 6.803569043 | -9.521794707 | 2.32E-14 | 5.94E-12 | 22.36269461 |
| 31 | RIF1 | -0.609310829 | 7.145838271 | -9.505108108 | 2.49E-14 | 6.10E-12 | 22.29345097 |
| 32 | SRFBP1 | -0.623582876 | 6.228571386 | -9.499014538 | 2.56E-14 | 6.10E-12 | 22.26816036 |
| 33 | DPY19L4 | -0.677660189 | 5.375468714 | -9.472529942 | 2.86E-14 | 6.46E-12 | 22.15821219 |
| 34 | IFT80 | -0.702582271 | 5.876259586 | -9.464680549 | 2.96E-14 | 6.59E-12 | 22.12561783 |
| 35 | RAD17 | -0.640319941 | 6.0772728 | -9.449759935 | 3.15E-14 | 6.76E-12 | 22.06364999 |
| 36 | USP1 | -0.748894622 | 6.901626971 | -9.435063742 | 3.36E-14 | 6.97E-12 | 22.00260092 |
| 37 | EIF5B | -0.594881341 | 7.260273443 | -9.433724634 | 3.38E-14 | 6.97E-12 | 21.99703752 |
| 38 | ZNF721 | -0.748319292 | 7.422178629 | -9.425559445 | 3.50E-14 | 7.05E-12 | 21.96311241 |
| 39 | ZMYM1 | -0.608184709 | 5.996545586 | -9.416967697 | 3.63E-14 | 7.12E-12 | 21.9274107 |
| 40 | GTF3C3 | -0.601118867 | 7.105358214 | -9.402180605 | 3.86E-14 | 7.28E-12 | 21.86595487 |
| 41 | HLTF | -0.610691835 | 5.687804714 | -9.379619607 | 4.25E-14 | 7.74E-12 | 21.77216551 |
| 42 | ABCE1 | -0.810906954 | 7.506517743 | -9.368857446 | 4.45E-14 | 7.95E-12 | 21.72741518 |
| 43 | IPO11 | -0.684024916 | 5.634112586 | -9.359999328 | 4.62E-14 | 8.09E-12 | 21.69057708 |
| 44 | DNAJC10 | -0.600756746 | 8.109352657 | -9.349158477 | 4.84E-14 | 8.30E-12 | 21.64548731 |
| 45 | SEC22A | -0.595971918 | 6.049435443 | -9.319105345 | 5.50E-14 | 8.96E-12 | 21.5204542 |
| 46 | PIGK | -0.75341271 | 5.631750257 | -9.293178083 | 6.15E-14 | 9.43E-12 | 21.41254605 |
| 47 | ZNF84 | -0.652184185 | 5.940821243 | -9.29275867 | 6.16E-14 | 9.43E-12 | 21.41080016 |
| 48 | PMS2 | -0.626942409 | 6.817134729 | -9.287880192 | 6.29E-14 | 9.46E-12 | 21.39049193 |
| 49 | CLK4 | -0.646201394 | 7.3691191 | -9.284150654 | 6.39E-14 | 9.52E-12 | 21.37496566 |
| 50 | BBS9 | -0.648494506 | 5.695179614 | -9.278292695 | 6.55E-14 | 9.60E-12 | 21.35057714 |
| 51 | MANEA | -0.668621839 | 5.886057214 | -9.195360828 | 9.33E-14 | 1.25E-11 | 21.00511229 |
| 52 | PREPL | -0.692541898 | 6.698558557 | -9.19345005 | 9.40E-14 | 1.25E-11 | 20.99714851 |
| 53 | DBT | -0.763758688 | 5.755404014 | -9.181378788 | 9.90E-14 | 1.28E-11 | 20.94683349 |
| 54 | RBM48 | -0.632074444 | 6.279850857 | -9.144694209 | 1.16E-13 | 1.46E-11 | 20.79388229 |
| 55 | DIPK1A | -0.768127099 | 6.1895158 | -9.142173106 | 1.17E-13 | 1.47E-11 | 20.78336852 |
| 56 | KPNA5 | -0.835546427 | 6.058428086 | -9.123402069 | 1.27E-13 | 1.58E-11 | 20.70507815 |
| 57 | LRRC40 | -0.687419556 | 6.204687643 | -9.067743093 | 1.61E-13 | 1.93E-11 | 20.47283989 |
| 58 | DDHD2 | -0.666204717 | 7.1282773 | -9.065751045 | 1.62E-13 | 1.93E-11 | 20.46452545 |
| 59 | SEPSECS | -0.70120034 | 6.2136485 | -9.013732676 | 2.03E-13 | 2.32E-11 | 20.24734991 |
| 60 | NEK1 | -0.588791109 | 5.572381486 | -9.008157251 | 2.08E-13 | 2.34E-11 | 20.22406586 |
| 61 | ZMAT1 | -0.72788432 | 5.612783643 | -9.006094983 | 2.09E-13 | 2.35E-11 | 20.21545311 |
| 62 | INTS7 | -0.680320946 | 6.242799771 | -8.9910794 | 2.23E-13 | 2.44E-11 | 20.15273757 |
| 63 | OSGEPL1 | -0.763083905 | 5.077504171 | -8.991069601 | 2.23E-13 | 2.44E-11 | 20.15269664 |
| 64 | TFB1M | -0.71861511 | 5.6453497 | -8.98591786 | 2.28E-13 | 2.46E-11 | 20.13117728 |
| 65 | N4BP2 | -0.74264143 | 6.351573429 | -8.983736759 | 2.30E-13 | 2.47E-11 | 20.12206627 |
| 66 | WRN | -0.633507959 | 5.460291 | -8.966032552 | 2.49E-13 | 2.60E-11 | 20.04810436 |
| 67 | SLC35A3 | -0.627644482 | 6.755271743 | -8.964758634 | 2.50E-13 | 2.60E-11 | 20.0427819 |
| 68 | ZNF141 | -0.771406418 | 6.843024757 | -8.96184 | 2.53E-13 | 2.61E-11 | 20.03058756 |
| 69 | MSH2 | -0.609938494 | 6.295195614 | -8.94378637 | 2.73E-13 | 2.77E-11 | 19.95515038 |
| 70 | PEX3 | -0.714550939 | 5.362519286 | -8.939977458 | 2.78E-13 | 2.80E-11 | 19.93923323 |
| 71 | SDAD1 | -0.820169036 | 7.9304199 | -8.938887232 | 2.79E-13 | 2.80E-11 | 19.93467716 |
| 72 | EEF1E1 | -0.631138854 | 4.7857941 | -8.915966033 | 3.08E-13 | 3.00E-11 | 19.83887887 |
| 73 | ZNF507 | -0.777434921 | 6.268961129 | -8.914815338 | 3.09E-13 | 3.00E-11 | 19.83406908 |
| 74 | METTL14 | -0.603347808 | 7.096497643 | -8.90318077 | 3.25E-13 | 3.06E-11 | 19.78543497 |
| 75 | SNX25 | -0.590702431 | 5.809873657 | -8.897790171 | 3.33E-13 | 3.10E-11 | 19.76289988 |
| 76 | NUP35 | -0.681792872 | 5.711426029 | -8.882303064 | 3.56E-13 | 3.30E-11 | 19.69815117 |
| 77 | ATG4C | -0.674505374 | 5.883568386 | -8.863832116 | 3.85E-13 | 3.51E-11 | 19.62091668 |
| 78 | WDR19 | -0.594658196 | 5.415459257 | -8.803486899 | 4.98E-13 | 4.36E-11 | 19.36851026 |
| 79 | POT1 | -0.599225478 | 5.605025429 | -8.800406977 | 5.05E-13 | 4.40E-11 | 19.35562475 |
| 80 | KIF3A | -0.8614125 | 5.1520718 | -8.780927393 | 5.49E-13 | 4.71E-11 | 19.27412114 |
| 81 | TMEM106B | -0.813204076 | 6.833510943 | -8.733452638 | 6.73E-13 | 5.47E-11 | 19.07543819 |
| 82 | ZNF146 | -0.6842192 | 6.686111043 | -8.732306591 | 6.76E-13 | 5.47E-11 | 19.07064118 |
| 83 | CROT | -0.632047365 | 5.750302186 | -8.727096684 | 6.92E-13 | 5.52E-11 | 19.04883364 |
| 84 | RBAK | -0.658156345 | 6.5278373 | -8.686991749 | 8.21E-13 | 6.32E-11 | 18.88093953 |
| 85 | DDX18 | -0.619149034 | 7.559404814 | -8.66352474 | 9.08E-13 | 6.77E-11 | 18.78267954 |
| 86 | SMC6 | -0.674478731 | 6.504008871 | -8.661037514 | 9.18E-13 | 6.82E-11 | 18.77226438 |
| 87 | GOLGA4 | -0.611469746 | 7.6523955 | -8.653532648 | 9.48E-13 | 6.97E-11 | 18.74083721 |
| 88 | NOP58 | -0.64446821 | 8.1871018 | -8.642389057 | 9.94E-13 | 7.20E-11 | 18.69417028 |
| 89 | KRIT1 | -0.605540909 | 6.5565355 | -8.634098318 | 1.03E-12 | 7.41E-11 | 18.6594487 |
| 90 | ASF1A | -0.823899018 | 5.764075286 | -8.616169285 | 1.11E-12 | 7.82E-11 | 18.58435701 |
| 91 | NDC1 | -0.595781996 | 5.458283443 | -8.608136263 | 1.15E-12 | 8.02E-11 | 18.55071037 |
| 92 | ABHD18 | -0.610647584 | 6.770743643 | -8.60505068 | 1.17E-12 | 8.07E-11 | 18.53778593 |
| 93 | MYNN | -0.654557458 | 6.447753814 | -8.597237328 | 1.21E-12 | 8.31E-11 | 18.50505768 |
| 94 | DZIP3 | -0.626342037 | 5.466725929 | -8.593823341 | 1.22E-12 | 8.35E-11 | 18.49075694 |
| 95 | PMS1 | -0.591653973 | 5.998302843 | -8.593388782 | 1.23E-12 | 8.35E-11 | 18.48893661 |
| 96 | TRMT61B | -0.618250407 | 5.676555986 | -8.587022881 | 1.26E-12 | 8.48E-11 | 18.46227004 |
| 97 | NAA15 | -0.640931311 | 7.0844408 | -8.540600562 | 1.54E-12 | 9.92E-11 | 18.26778633 |
| 98 | ERO1B | -0.607482772 | 5.416107914 | -8.535160765 | 1.58E-12 | 1.01E-10 | 18.24499423 |
| 99 | MTX2 | -0.602779929 | 5.514660729 | -8.521806396 | 1.67E-12 | 1.05E-10 | 18.18903905 |
| 100 | WDR89 | -0.678399023 | 6.528642829 | -8.504000719 | 1.80E-12 | 1.12E-10 | 18.11442865 |
| 101 | ALG6 | -0.687153069 | 5.971391129 | -8.499231626 | 1.84E-12 | 1.13E-10 | 18.09444415 |
| 102 | GPR171 | -1.055939301 | 6.050270057 | -8.456366811 | 2.21E-12 | 1.30E-10 | 17.91480947 |
| 103 | GNPDA2 | -0.6457612 | 5.306803843 | -8.450939624 | 2.26E-12 | 1.32E-10 | 17.89206407 |
| 104 | SUCLG2 | -0.721077842 | 8.064207929 | -8.438454314 | 2.39E-12 | 1.38E-10 | 17.83973681 |
| 105 | PGM3 | -0.598906972 | 5.222268886 | -8.426378251 | 2.51E-12 | 1.44E-10 | 17.78912327 |
| 106 | AGPAT5 | -0.600505977 | 6.715661243 | -8.425294889 | 2.52E-12 | 1.44E-10 | 17.78458258 |
| 107 | ACTR6 | -0.760623746 | 5.611188671 | -8.403681465 | 2.77E-12 | 1.56E-10 | 17.69399227 |
| 108 | PHOSPHO2 | -0.895195114 | 4.355366014 | -8.381778997 | 3.04E-12 | 1.67E-10 | 17.60218671 |
| 109 | CWF19L2 | -0.60462245 | 6.6948526 | -8.380532939 | 3.06E-12 | 1.68E-10 | 17.59696368 |
| 110 | CD200R1 | -0.897289334 | 5.280295871 | -8.375305421 | 3.13E-12 | 1.70E-10 | 17.57505169 |
| 111 | CCNC | -0.591472449 | 7.415479557 | -8.370123821 | 3.20E-12 | 1.72E-10 | 17.553332 |
| 112 | CEP85L | -0.695975486 | 7.291523243 | -8.367850394 | 3.23E-12 | 1.74E-10 | 17.54380244 |
| 113 | RPE | -0.620039693 | 7.857508414 | -8.359361859 | 3.35E-12 | 1.80E-10 | 17.50822065 |
| 114 | ACADSB | -0.676274546 | 5.8162102 | -8.356081695 | 3.40E-12 | 1.81E-10 | 17.49447093 |
| 115 | ICE2 | -0.595949697 | 6.9686872 | -8.350357874 | 3.48E-12 | 1.84E-10 | 17.47047782 |
| 116 | EPHA4 | -0.639706155 | 6.203885686 | -8.34820333 | 3.51E-12 | 1.85E-10 | 17.46144636 |
| 117 | GCNT4 | -1.118042663 | 5.975331229 | -8.317845725 | 4.00E-12 | 2.06E-10 | 17.33419089 |
| 118 | DNAH14 | -0.893087512 | 5.272830086 | -8.278430853 | 4.74E-12 | 2.36E-10 | 17.16896519 |
| 119 | SENP7 | -0.694054307 | 7.070969757 | -8.271322525 | 4.89E-12 | 2.42E-10 | 17.13916722 |
| 120 | CSNK1G3 | -0.637426916 | 6.7954661 | -8.262569203 | 5.07E-12 | 2.48E-10 | 17.1024735 |
| 121 | FARS2 | -0.600810228 | 6.595776214 | -8.25576866 | 5.23E-12 | 2.53E-10 | 17.07396583 |
| 122 | PRIM2 | -0.708850675 | 5.360550029 | -8.250916136 | 5.34E-12 | 2.58E-10 | 17.05362424 |
| 123 | SSR3 | -0.638716337 | 7.292325729 | -8.249478785 | 5.37E-12 | 2.59E-10 | 17.04759893 |
| 124 | SPDL1 | -0.595370731 | 4.646667957 | -8.241332322 | 5.56E-12 | 2.65E-10 | 17.01344938 |
| 125 | ARHGAP15 | -0.625967056 | 8.714762657 | -8.221005156 | 6.07E-12 | 2.83E-10 | 16.92823974 |
| 126 | EMC2 | -0.654889331 | 6.428137271 | -8.216565321 | 6.18E-12 | 2.87E-10 | 16.90962853 |
| 127 | IBTK | -0.640992357 | 7.373237114 | -8.183519566 | 7.13E-12 | 3.23E-10 | 16.7711079 |
| 128 | SERINC1 | -0.587755172 | 9.366819643 | -8.165067701 | 7.71E-12 | 3.44E-10 | 16.69376444 |
| 129 | ELMOD2 | -0.616446629 | 5.475177543 | -8.162333977 | 7.80E-12 | 3.46E-10 | 16.68230587 |
| 130 | VPS50 | -0.647666405 | 5.297652257 | -8.135134073 | 8.77E-12 | 3.83E-10 | 16.56829914 |
| 131 | TAF1B | -0.609939196 | 5.481927157 | -8.131433026 | 8.91E-12 | 3.88E-10 | 16.55278693 |
| 132 | COX20 | -0.666083436 | 7.580006243 | -8.116690097 | 9.49E-12 | 4.08E-10 | 16.4909962 |
| 133 | MGAT4A | -0.808161947 | 7.678764857 | -8.103467852 | 1.00E-11 | 4.24E-10 | 16.43558092 |
| 134 | ORC3 | -0.646382308 | 5.838920714 | -8.094770558 | 1.04E-11 | 4.36E-10 | 16.39913109 |
| 135 | NBEAL1 | -0.585483976 | 5.009866257 | -8.094454904 | 1.04E-11 | 4.36E-10 | 16.39780822 |
| 136 | GCC2 | -0.741966965 | 7.157191114 | -8.081145637 | 1.11E-11 | 4.55E-10 | 16.34203172 |
| 137 | ACTN1 | 0.644023038 | 9.0408177 | 8.078972615 | 1.12E-11 | 4.57E-10 | 16.33292523 |
| 138 | KRCC1 | -0.655356955 | 6.310587786 | -8.058488792 | 1.22E-11 | 4.89E-10 | 16.24708677 |
| 139 | HIVEP2 | -0.8191479 | 6.802773371 | -8.054391933 | 1.24E-11 | 4.95E-10 | 16.22991939 |
| 140 | ORMDL1 | -0.611692386 | 7.745724857 | -8.036330207 | 1.34E-11 | 5.26E-10 | 16.15423702 |
| 141 | GIMAP2 | -0.697622201 | 7.9409135 | -8.003408308 | 1.54E-11 | 5.83E-10 | 16.01630125 |
| 142 | CEP135 | -0.650166344 | 5.481702786 | -7.999927866 | 1.57E-11 | 5.88E-10 | 16.00172006 |
| 143 | UFSP2 | -0.597561245 | 5.036484114 | -7.99369656 | 1.61E-11 | 6.00E-10 | 15.97561479 |
| 144 | USP40 | -0.609716855 | 5.568844243 | -7.991586792 | 1.62E-11 | 6.01E-10 | 15.96677634 |
| 145 | PIBF1 | -0.615685753 | 5.733851814 | -7.989671198 | 1.64E-11 | 6.04E-10 | 15.95875143 |
| 146 | PPM1K | -0.694842448 | 7.027362086 | -7.975965194 | 1.74E-11 | 6.26E-10 | 15.90133555 |
| 147 | CSPP1 | -0.589614734 | 5.655956686 | -7.974646816 | 1.75E-11 | 6.26E-10 | 15.89581293 |
| 148 | ZNF383 | -0.660020213 | 6.293600729 | -7.974381691 | 1.75E-11 | 6.26E-10 | 15.89470234 |
| 149 | AK9 | -0.738936909 | 5.722010757 | -7.948430969 | 1.95E-11 | 6.88E-10 | 15.78600356 |
| 150 | TRAF5 | -0.649368966 | 6.895098857 | -7.91887802 | 2.22E-11 | 7.70E-10 | 15.66223462 |
| 151 | MRPL1 | -0.645658136 | 6.067271829 | -7.903167077 | 2.37E-11 | 8.17E-10 | 15.59644511 |
| 152 | SLAMF6 | -0.898717503 | 8.045564471 | -7.880467354 | 2.62E-11 | 8.78E-10 | 15.50140134 |
| 153 | KIAA1586 | -0.594747879 | 5.971914014 | -7.87042243 | 2.73E-11 | 9.06E-10 | 15.45934765 |
| 154 | LSM8 | -0.668018947 | 6.7852022 | -7.865921926 | 2.78E-11 | 9.22E-10 | 15.44050692 |
| 155 | OXNAD1 | -0.839579388 | 7.317991143 | -7.824523468 | 3.32E-11 | 1.05E-09 | 15.26722582 |
| 156 | PNPT1 | -0.715159673 | 6.827036 | -7.812014181 | 3.51E-11 | 1.09E-09 | 15.21487615 |
| 157 | LARP7 | -0.621460693 | 5.9457125 | -7.809333716 | 3.55E-11 | 1.10E-09 | 15.20365942 |
| 158 | TMEM243 | -0.609075261 | 8.094183343 | -7.748591123 | 4.60E-11 | 1.36E-09 | 14.94954 |
| 159 | FASTKD1 | -0.637455824 | 6.023001229 | -7.741713934 | 4.74E-11 | 1.39E-09 | 14.92077727 |
| 160 | TXNDC9 | -0.591835931 | 5.815796543 | -7.736100095 | 4.86E-11 | 1.41E-09 | 14.89729961 |
| 161 | ITK | -0.848030421 | 8.887027529 | -7.703125578 | 5.59E-11 | 1.60E-09 | 14.75942136 |
| 162 | PPIA | -1.031843063 | 6.640441971 | -7.694819516 | 5.80E-11 | 1.65E-09 | 14.72469759 |
| 163 | PARP15 | -1.007588715 | 7.103473057 | -7.68514431 | 6.04E-11 | 1.69E-09 | 14.68425365 |
| 164 | EXOSC8 | -0.647060782 | 5.470715343 | -7.6542757 | 6.90E-11 | 1.89E-09 | 14.55524443 |
| 165 | TBC1D19 | -0.673839724 | 5.677334671 | -7.641876727 | 7.27E-11 | 1.97E-09 | 14.50343715 |
| 166 | ODF2L | -0.690784163 | 5.889316843 | -7.639696647 | 7.34E-11 | 1.99E-09 | 14.49432872 |
| 167 | DCAF16 | -0.659146252 | 7.354077386 | -7.638529426 | 7.38E-11 | 1.99E-09 | 14.48945214 |
| 168 | NUP43 | -0.609760511 | 7.580243986 | -7.634601861 | 7.50E-11 | 2.02E-09 | 14.47304343 |
| 169 | LRIF1 | -0.801062603 | 5.140431943 | -7.612744322 | 8.24E-11 | 2.17E-09 | 14.3817394 |
| 170 | STAMBPL1 | -0.633887534 | 6.474584871 | -7.57725224 | 9.59E-11 | 2.48E-09 | 14.23352959 |
| 171 | ZNF611 | -0.597158202 | 5.589413086 | -7.573936688 | 9.72E-11 | 2.51E-09 | 14.21968751 |
| 172 | SRSF3 | -0.688578885 | 8.535933543 | -7.566778683 | 1.00E-10 | 2.56E-09 | 14.18980552 |
| 173 | P2RY10 | -0.863650096 | 5.859762657 | -7.563377987 | 1.02E-10 | 2.58E-09 | 14.17560981 |
| 174 | TMEM156 | -0.880747329 | 6.533473243 | -7.559560532 | 1.03E-10 | 2.61E-09 | 14.1596751 |
| 175 | MRPS14 | -0.690086649 | 6.293605814 | -7.543457715 | 1.11E-10 | 2.76E-09 | 14.0924676 |
| 176 | ERI1 | -0.63131742 | 4.862657643 | -7.521989833 | 1.21E-10 | 2.96E-09 | 14.00288973 |
| 177 | AKT3 | -0.728302449 | 6.861134271 | -7.510339041 | 1.28E-10 | 3.06E-09 | 13.95428567 |
| 178 | YAE1 | -0.628228707 | 5.340134957 | -7.507001906 | 1.29E-10 | 3.08E-09 | 13.9403654 |
| 179 | TMTC3 | -0.654651785 | 5.457533943 | -7.500760777 | 1.33E-10 | 3.14E-09 | 13.91433333 |
| 180 | ZNF680 | -0.640593885 | 6.118836857 | -7.473031231 | 1.50E-10 | 3.43E-09 | 13.79869896 |
| 181 | CAMK2D | -0.644482465 | 7.284710371 | -7.459439602 | 1.59E-10 | 3.59E-09 | 13.74203728 |
| 182 | LDAH | -0.645465873 | 5.957128243 | -7.45775883 | 1.60E-10 | 3.60E-09 | 13.73503113 |
| 183 | TPMT | -1.092527251 | 6.216106771 | -7.451419963 | 1.64E-10 | 3.68E-09 | 13.70860968 |
| 184 | HAT1 | -0.609133596 | 6.592996214 | -7.430184092 | 1.80E-10 | 3.97E-09 | 13.62011297 |
| 185 | MRPS10 | -0.973306897 | 6.5133603 | -7.423553378 | 1.85E-10 | 4.06E-09 | 13.5924864 |
| 186 | XRCC4 | -0.586198296 | 6.597793943 | -7.387704673 | 2.16E-10 | 4.54E-09 | 13.44317315 |
| 187 | G2E3 | -0.613407483 | 5.840044343 | -7.371638873 | 2.31E-10 | 4.82E-09 | 13.37628497 |
| 188 | UBE2V2 | -0.714320216 | 6.675605186 | -7.363789304 | 2.39E-10 | 4.95E-09 | 13.34361048 |
| 189 | CSTF3 | -0.592902898 | 6.332757929 | -7.348048019 | 2.55E-10 | 5.17E-09 | 13.2780988 |
| 190 | DPP4 | -0.936891801 | 6.352979471 | -7.333444061 | 2.72E-10 | 5.47E-09 | 13.21733584 |
| 191 | CD96 | -0.701316472 | 8.584845171 | -7.319243493 | 2.89E-10 | 5.78E-09 | 13.15826578 |
| 192 | PATJ | -0.761855353 | 6.402569786 | -7.292405082 | 3.24E-10 | 6.37E-09 | 13.04666608 |
| 193 | C8orf44 | -0.587001871 | 5.488037086 | -7.277905644 | 3.44E-10 | 6.66E-09 | 12.98639662 |
| 194 | HIBCH | -0.648041804 | 6.786443786 | -7.276850159 | 3.46E-10 | 6.66E-09 | 12.98200994 |
| 195 | ESF1 | -0.602519734 | 5.894641043 | -7.264552344 | 3.64E-10 | 6.94E-09 | 12.93090538 |
| 196 | CLU | 0.608446437 | 9.120261157 | 7.196196282 | 4.88E-10 | 8.76E-09 | 12.64706317 |
| 197 | PLEKHA3 | -0.754143109 | 6.521872671 | -7.194095737 | 4.92E-10 | 8.82E-09 | 12.63834686 |
| 198 | SAMD9 | -0.6089784 | 7.733881657 | -7.169755922 | 5.46E-10 | 9.58E-09 | 12.53737464 |
| 199 | COPS4 | -0.62397934 | 5.961633743 | -7.162108338 | 5.64E-10 | 9.80E-09 | 12.50565951 |
| 200 | ZNF701 | -0.773850399 | 6.692708986 | -7.151599785 | 5.89E-10 | 1.01E-08 | 12.46208795 |
| 201 | GSN | 0.585297448 | 8.231012843 | 7.148779147 | 5.97E-10 | 1.02E-08 | 12.45039439 |
| 202 | SLC25A40 | -0.606093885 | 6.021334 | -7.148210142 | 5.98E-10 | 1.02E-08 | 12.44803554 |
| 203 | TRAT1 | -1.00691452 | 8.493817057 | -7.126457578 | 6.56E-10 | 1.10E-08 | 12.35788024 |
| 204 | AP3M2 | -0.627648577 | 6.915996629 | -7.111339037 | 6.99E-10 | 1.16E-08 | 12.29524509 |
| 205 | RAB28 | -0.675654845 | 5.240934786 | -7.100409422 | 7.33E-10 | 1.21E-08 | 12.24997731 |
| 206 | ICOS | -0.893473906 | 6.957816071 | -7.090418817 | 7.64E-10 | 1.24E-08 | 12.20860829 |
| 207 | FPGT | -0.614775387 | 6.250483886 | -7.084948601 | 7.82E-10 | 1.26E-08 | 12.18596119 |
| 208 | CLEC2D | -0.723739568 | 9.207255914 | -7.055589117 | 8.86E-10 | 1.40E-08 | 12.06445885 |
| 209 | ABCD2 | -0.932702531 | 6.5916581 | -7.055193094 | 8.88E-10 | 1.40E-08 | 12.06282049 |
| 210 | NR1D2 | -0.686025482 | 7.390093357 | -7.0339073 | 9.72E-10 | 1.51E-08 | 11.97478311 |
| 211 | LSM5 | -0.656231227 | 4.348521357 | -7.027661798 | 9.98E-10 | 1.54E-08 | 11.94896031 |
| 212 | IFT57 | -0.640910339 | 5.337714843 | -6.974559598 | 1.25E-09 | 1.83E-08 | 11.72956077 |
| 213 | SLC18B1 | -0.612693344 | 6.363446657 | -6.967889036 | 1.29E-09 | 1.87E-08 | 11.70202078 |
| 214 | MCUB | -0.625422178 | 8.487481243 | -6.9396086 | 1.45E-09 | 2.07E-08 | 11.58531458 |
| 215 | BIRC3 | -0.855629621 | 8.611556571 | -6.926518957 | 1.53E-09 | 2.17E-08 | 11.53132586 |
| 216 | USP53 | -0.883197472 | 6.792882071 | -6.878758469 | 1.87E-09 | 2.55E-08 | 11.3344954 |
| 217 | TRIP11 | -0.666224551 | 6.636435743 | -6.864602563 | 1.99E-09 | 2.68E-08 | 11.27620527 |
| 218 | ZNF14 | -0.621627671 | 5.614518257 | -6.86153847 | 2.02E-09 | 2.71E-08 | 11.26359119 |
| 219 | PLEKHA1 | -0.626540701 | 7.689128743 | -6.860620893 | 2.02E-09 | 2.72E-08 | 11.25981398 |
| 220 | TRAJ17 | -0.630549571 | 6.331548371 | -6.86045467 | 2.03E-09 | 2.72E-08 | 11.25912973 |
| 221 | RECQL | -0.58735777 | 7.533165043 | -6.852178023 | 2.10E-09 | 2.79E-08 | 11.22506336 |
| 222 | COMMD8 | -0.684105932 | 6.011227871 | -6.819441129 | 2.41E-09 | 3.13E-08 | 11.09039795 |
| 223 | KRR1 | -0.592512198 | 6.715945371 | -6.810260161 | 2.50E-09 | 3.24E-08 | 11.05265421 |
| 224 | ZNF649 | -0.604545403 | 5.226225271 | -6.809485202 | 2.51E-09 | 3.25E-08 | 11.04946875 |
| 225 | ZNF382 | -0.680401024 | 4.309008814 | -6.793126041 | 2.69E-09 | 3.42E-08 | 10.98224156 |
| 226 | DOCK9 | -0.623578581 | 6.852997414 | -6.775931816 | 2.89E-09 | 3.62E-08 | 10.91161789 |
| 227 | TAS2R20 | -0.677801732 | 6.893322329 | -6.773020282 | 2.93E-09 | 3.65E-08 | 10.89966264 |
| 228 | GCSAM | -0.651159002 | 6.391169043 | -6.772169054 | 2.94E-09 | 3.66E-08 | 10.89616755 |
| 229 | MPIG6B | 0.612593927 | 7.266758043 | 6.753129514 | 3.19E-09 | 3.90E-08 | 10.81801606 |
| 230 | TRAV8-3 | -0.745523724 | 5.412808314 | -6.729004813 | 3.53E-09 | 4.21E-08 | 10.71905724 |
| 231 | MTARC2 | 0.648911028 | 6.496767114 | 6.726470166 | 3.56E-09 | 4.25E-08 | 10.70866451 |
| 232 | PTPN22 | -0.587460458 | 7.023495086 | -6.721036709 | 3.65E-09 | 4.34E-08 | 10.68638868 |
| 233 | SLC25A36 | -0.622094933 | 6.069336586 | -6.694884694 | 4.07E-09 | 4.75E-08 | 10.57922557 |
| 234 | GBP4 | -0.634332417 | 7.308983971 | -6.682053891 | 4.30E-09 | 4.96E-08 | 10.52668172 |
| 235 | IL7R | -0.926897558 | 10.18316893 | -6.67865337 | 4.36E-09 | 5.02E-08 | 10.5127598 |
| 236 | GZMK | -1.104623552 | 8.122423171 | -6.672989867 | 4.46E-09 | 5.12E-08 | 10.48957655 |
| 237 | SLAMF1 | -0.586142196 | 5.630308814 | -6.659009205 | 4.73E-09 | 5.36E-08 | 10.43236594 |
| 238 | NMT2 | -0.641238244 | 6.3381669 | -6.654525572 | 4.82E-09 | 5.45E-08 | 10.41402396 |
| 239 | CRYZ | -0.756316616 | 5.2836269 | -6.650067276 | 4.91E-09 | 5.53E-08 | 10.39578836 |
| 240 | COX11 | -0.59795708 | 6.175955714 | -6.633998777 | 5.26E-09 | 5.85E-08 | 10.33008659 |
| 241 | CAMK4 | -0.889385515 | 7.268287557 | -6.607755731 | 5.87E-09 | 6.38E-08 | 10.22285967 |
| 242 | RWDD4 | -0.736689558 | 5.897320871 | -6.582653311 | 6.52E-09 | 6.97E-08 | 10.1203843 |
| 243 | ANAPC10 | -0.676345467 | 5.665200729 | -6.574984511 | 6.73E-09 | 7.13E-08 | 10.08909605 |
| 244 | RALGAPA1 | -0.87269348 | 5.193599286 | -6.524127611 | 8.33E-09 | 8.51E-08 | 9.881820797 |
| 245 | STAT4 | -0.656740365 | 7.909075743 | -6.484321062 | 9.84E-09 | 9.79E-08 | 9.719853629 |
| 246 | LEF1 | -0.989025357 | 8.931927 | -6.465239203 | 1.07E-08 | 1.05E-07 | 9.642298802 |
| 247 | LYRM7 | -0.640341402 | 5.813884057 | -6.449857245 | 1.14E-08 | 1.10E-07 | 9.579823112 |
| 248 | ZNF627 | -0.624239347 | 5.114976943 | -6.443520301 | 1.17E-08 | 1.13E-07 | 9.554095736 |
| 249 | TAS2R4 | -1.148425245 | 4.973663814 | -6.441939323 | 1.17E-08 | 1.13E-07 | 9.547678117 |
| 250 | ATF7IP2 | -0.647438861 | 6.271864629 | -6.437288729 | 1.20E-08 | 1.15E-07 | 9.5288024 |
| 251 | ITGA6 | -0.714087249 | 7.182051114 | -6.419265692 | 1.29E-08 | 1.22E-07 | 9.455683743 |
| 252 | MMADHC | -0.702220972 | 6.818035386 | -6.408954638 | 1.35E-08 | 1.26E-07 | 9.413875887 |
| 253 | ELANE | 0.637975752 | 7.231421943 | 6.406998579 | 1.36E-08 | 1.27E-07 | 9.405946679 |
| 254 | RBM43 | -0.590040592 | 5.045429729 | -6.383002907 | 1.50E-08 | 1.38E-07 | 9.308727417 |
| 255 | ZNF675 | -0.981576668 | 4.747795229 | -6.3705524 | 1.58E-08 | 1.43E-07 | 9.258321374 |
| 256 | PSMA5 | -0.606689957 | 7.184204714 | -6.363217642 | 1.63E-08 | 1.47E-07 | 9.22863865 |
| 257 | DDX60 | -0.766188044 | 6.471303814 | -6.351725112 | 1.71E-08 | 1.53E-07 | 9.182148258 |
| 258 | NELL2 | -1.081966735 | 7.462273671 | -6.30162774 | 2.11E-08 | 1.81E-07 | 8.979754949 |
| 259 | SSX8P | 0.674250991 | 8.1997811 | 6.299806456 | 2.12E-08 | 1.82E-07 | 8.972405172 |
| 260 | KMO | -0.773124726 | 6.148719586 | -6.273950668 | 2.36E-08 | 2.00E-07 | 8.868127586 |
| 261 | PYROXD1 | -0.614143845 | 6.409827457 | -6.269307492 | 2.41E-08 | 2.03E-07 | 8.849414039 |
| 262 | PGAP1 | -0.696070568 | 4.624242429 | -6.253285028 | 2.57E-08 | 2.15E-07 | 8.784867922 |
| 263 | ZNF468 | -0.588602301 | 5.962634614 | -6.250455088 | 2.61E-08 | 2.17E-07 | 8.773472393 |
| 264 | CTTN | 0.615179523 | 7.5359506 | 6.227835524 | 2.86E-08 | 2.36E-07 | 8.682440996 |
| 265 | ARHGDIA | 0.639189752 | 7.6115952 | 6.22167596 | 2.93E-08 | 2.42E-07 | 8.657668376 |
| 266 | ZNF138 | -0.591709157 | 5.681654186 | -6.201938936 | 3.18E-08 | 2.58E-07 | 8.578337135 |
| 267 | EIF4E | -0.840137759 | 5.138610771 | -6.179587852 | 3.49E-08 | 2.78E-07 | 8.488587018 |
| 268 | CD28 | -0.73230479 | 7.290721186 | -6.176441156 | 3.54E-08 | 2.81E-07 | 8.475959144 |
| 269 | TXK | -0.835054716 | 8.049569071 | -6.156284601 | 3.85E-08 | 3.01E-07 | 8.395114544 |
| 270 | VSIG1 | -0.643195395 | 5.546770743 | -6.145198698 | 4.03E-08 | 3.13E-07 | 8.350684137 |
| 271 | EIF3E | -0.638224602 | 7.3641967 | -6.091360499 | 5.03E-08 | 3.77E-07 | 8.135251945 |
| 272 | PPIL3 | -0.598206728 | 6.832555329 | -6.064837693 | 5.60E-08 | 4.14E-07 | 8.029334187 |
| 273 | IKZF2 | -0.636464099 | 6.0018891 | -6.042609335 | 6.14E-08 | 4.48E-07 | 7.940676479 |
| 274 | TYW1B | -0.653956627 | 3.888930029 | -6.020210179 | 6.73E-08 | 4.83E-07 | 7.851440803 |
| 275 | LPAR6 | -0.666676675 | 7.282694143 | -6.001134917 | 7.28E-08 | 5.16E-07 | 7.7755299 |
| 276 | STRBP | -0.692254968 | 5.148333486 | -5.992166417 | 7.55E-08 | 5.33E-07 | 7.739865904 |
| 277 | CCDC141 | -0.62029553 | 5.541933571 | -5.984081773 | 7.81E-08 | 5.48E-07 | 7.70773131 |
| 278 | DNTTIP2 | -0.62663948 | 6.836558829 | -5.980739414 | 7.91E-08 | 5.54E-07 | 7.694450285 |
| 279 | RPL22 | -0.585470531 | 8.767363257 | -5.9548877 | 8.80E-08 | 6.04E-07 | 7.591808463 |
| 280 | PWAR6 | -0.919856171 | 3.394218171 | -5.952291481 | 8.89E-08 | 6.09E-07 | 7.581508418 |
| 281 | SKAP1 | -0.612097254 | 7.533555229 | -5.946242469 | 9.11E-08 | 6.20E-07 | 7.557515715 |
| 282 | KIAA0232 | -0.649780804 | 5.399578029 | -5.917917606 | 1.02E-07 | 6.85E-07 | 7.445275332 |
| 283 | AASDH | -0.717701016 | 5.663169 | -5.913537065 | 1.04E-07 | 6.95E-07 | 7.427932803 |
| 284 | RHOH | -0.625041148 | 7.9706027 | -5.912117012 | 1.05E-07 | 6.98E-07 | 7.422311744 |
| 285 | RCAN3 | -0.646180196 | 8.3456763 | -5.905378008 | 1.08E-07 | 7.14E-07 | 7.395642565 |
| 286 | TESPA1 | -0.617163812 | 7.693482543 | -5.88446341 | 1.17E-07 | 7.69E-07 | 7.312939293 |
| 287 | ZNF417 | -0.631488306 | 6.866730986 | -5.850215392 | 1.35E-07 | 8.68E-07 | 7.177725751 |
| 288 | ZNF718 | -0.641937696 | 5.040404786 | -5.845697012 | 1.37E-07 | 8.82E-07 | 7.159906988 |
| 289 | MYL9 | 0.722009924 | 8.553924914 | 5.794926802 | 1.69E-07 | 1.05E-06 | 6.960016442 |
| 290 | SAMD12 | -0.743645263 | 4.702462143 | -5.784600641 | 1.76E-07 | 1.09E-06 | 6.919435234 |
| 291 | GOLGA8B | -0.750858804 | 9.0294448 | -5.744588868 | 2.07E-07 | 1.25E-06 | 6.762433795 |
| 292 | EIF1AX | -0.641920404 | 6.719948386 | -5.743722278 | 2.08E-07 | 1.25E-06 | 6.759037706 |
| 293 | HINT1 | -0.715733966 | 8.190312929 | -5.704356989 | 2.43E-07 | 1.43E-06 | 6.604963803 |
| 294 | GP1BA | 0.593042382 | 7.4019135 | 5.702231192 | 2.46E-07 | 1.44E-06 | 6.59665449 |
| 295 | MSMO1 | -0.610292663 | 5.413754657 | -5.656624298 | 2.95E-07 | 1.69E-06 | 6.418660978 |
| 296 | CCDC127 | -0.661797247 | 5.290812743 | -5.656191665 | 2.96E-07 | 1.69E-06 | 6.416975044 |
| 297 | THEM4 | -0.646731961 | 5.388053871 | -5.641755868 | 3.13E-07 | 1.78E-06 | 6.360747531 |
| 298 | ATAD2B | -0.724120317 | 5.417370914 | -5.627613547 | 3.32E-07 | 1.87E-06 | 6.305715353 |
| 299 | CBWD3 | -0.620492383 | 6.899025014 | -5.568222125 | 4.21E-07 | 2.29E-06 | 6.075178047 |
| 300 | TIGD1 | -0.666469123 | 4.645672943 | -5.564708341 | 4.27E-07 | 2.32E-06 | 6.061568134 |
| 301 | LYPLAL1 | -0.644479122 | 5.608356614 | -5.564461254 | 4.27E-07 | 2.32E-06 | 6.060611218 |
| 302 | PYHIN1 | -0.638230939 | 7.622193 | -5.562544357 | 4.31E-07 | 2.33E-06 | 6.053188041 |
| 303 | TC2N | -0.627607158 | 9.203318186 | -5.52261119 | 5.05E-07 | 2.68E-06 | 5.898773541 |
| 304 | HMGN1 | 0.5948366 | 7.2317353 | 5.498788187 | 5.55E-07 | 2.91E-06 | 5.806862377 |
| 305 | SEPTIN7 | -0.619049104 | 7.575344429 | -5.490555443 | 5.74E-07 | 2.99E-06 | 5.77513637 |
| 306 | KRTAP10-7 | 0.629408702 | 6.8359383 | 5.468489857 | 6.27E-07 | 3.23E-06 | 5.690197192 |
| 307 | MCOLN2 | -0.646073589 | 6.555928443 | -5.439956013 | 7.02E-07 | 3.55E-06 | 5.580563231 |
| 308 | CD58 | -0.695404688 | 6.055378129 | -5.42043115 | 7.58E-07 | 3.79E-06 | 5.505678158 |
| 309 | TAS2R46 | -0.932074424 | 3.188453114 | -5.414910309 | 7.75E-07 | 3.86E-06 | 5.484523637 |
| 310 | ITGA2B | 0.675304141 | 9.001321943 | 5.365621544 | 9.42E-07 | 4.56E-06 | 5.29605558 |
| 311 | SNRPD1 | -0.590573661 | 5.002628 | -5.363189147 | 9.51E-07 | 4.60E-06 | 5.28677324 |
| 312 | MPP1 | 0.59105438 | 9.811542071 | 5.358740861 | 9.68E-07 | 4.67E-06 | 5.269802553 |
| 313 | BZW1 | -0.593261716 | 8.009800086 | -5.334992006 | 1.06E-06 | 5.06E-06 | 5.179298229 |
| 314 | PTGS1 | 0.627575062 | 8.498722271 | 5.331632955 | 1.08E-06 | 5.12E-06 | 5.166510926 |
| 315 | DNAJB4 | -0.59007 | 5.391768571 | -5.326995668 | 1.10E-06 | 5.20E-06 | 5.148863209 |
| 316 | LRRN3 | -0.908384494 | 5.391051914 | -5.321517547 | 1.12E-06 | 5.30E-06 | 5.128023986 |
| 317 | SUMO4 | -0.754638716 | 4.581112671 | -5.287671256 | 1.28E-06 | 5.96E-06 | 4.99947269 |
| 318 | TAS2R5 | -0.696580621 | 4.259920043 | -5.251793271 | 1.47E-06 | 6.71E-06 | 4.86359052 |
| 319 | ZNF107 | -0.590978449 | 6.3950931 | -5.194829435 | 1.84E-06 | 8.18E-06 | 4.648680715 |
| 320 | ITGB3 | 0.758395393 | 10.1055281 | 5.169515199 | 2.03E-06 | 8.91E-06 | 4.553510219 |
| 321 | FCMR | -0.592818786 | 8.814047186 | -5.165781008 | 2.06E-06 | 9.02E-06 | 4.539488889 |
| 322 | TAF9 | -0.653313016 | 5.462217371 | -5.144666716 | 2.24E-06 | 9.70E-06 | 4.460293664 |
| 323 | FUNDC2 | -0.708432668 | 4.713920586 | -5.124095672 | 2.43E-06 | 1.04E-05 | 4.383277228 |
| 324 | S1PR1 | -0.590606506 | 8.549342843 | -5.079123342 | 2.89E-06 | 1.21E-05 | 4.215395872 |
| 325 | DYSF | 0.593023432 | 7.845046214 | 5.066064552 | 3.04E-06 | 1.26E-05 | 4.166775401 |
| 326 | DENND11 | -0.721740218 | 5.795979557 | -5.044279435 | 3.30E-06 | 1.36E-05 | 4.085794713 |
| 327 | ZC2HC1A | -0.592637853 | 5.7346403 | -5.018486604 | 3.65E-06 | 1.48E-05 | 3.990127797 |
| 328 | LCE1D | 0.964776665 | 7.099916514 | 5.014382338 | 3.71E-06 | 1.50E-05 | 3.974926167 |
| 329 | PADI4 | 0.663315368 | 7.842853771 | 4.994740983 | 4.00E-06 | 1.61E-05 | 3.902258891 |
| 330 | FCRL3 | -0.622571305 | 7.013111214 | -4.945935816 | 4.82E-06 | 1.89E-05 | 3.72228435 |
| 331 | CD9 | 0.6103651 | 8.209066957 | 4.943964663 | 4.86E-06 | 1.91E-05 | 3.715033369 |
| 332 | HERC6 | -0.589060257 | 6.576489486 | -4.938386119 | 4.96E-06 | 1.94E-05 | 3.694520006 |
| 333 | AIDA | -0.607393046 | 3.151362686 | -4.90895391 | 5.55E-06 | 2.14E-05 | 3.58647862 |
| 334 | PLCL1 | -0.594928056 | 5.072454843 | -4.907394272 | 5.59E-06 | 2.15E-05 | 3.580762204 |
| 335 | CD2 | -0.612467918 | 9.2331677 | -4.806549035 | 8.19E-06 | 3.02E-05 | 3.213055563 |
| 336 | THBS1 | 0.716789423 | 8.943342571 | 4.704223149 | 1.20E-05 | 4.26E-05 | 2.843913049 |
| 337 | YBX3 | 0.587401599 | 9.0147707 | 4.681841927 | 1.31E-05 | 4.59E-05 | 2.763721008 |
| 338 | NOG | -0.740595269 | 5.777284871 | -4.660646529 | 1.42E-05 | 4.91E-05 | 2.687962916 |
| 339 | IL2RA | -0.590393549 | 6.936413286 | -4.650782469 | 1.47E-05 | 5.08E-05 | 2.652767942 |
| 340 | PGLYRP1 | 1.04140441 | 6.707069 | 4.630010386 | 1.59E-05 | 5.45E-05 | 2.578782483 |
| 341 | ITGB3BP | -0.597421828 | 5.857722657 | -4.568284428 | 2.00E-05 | 6.70E-05 | 2.359976291 |
| 342 | MMP25 | 0.62198102 | 7.036267686 | 4.520988169 | 2.37E-05 | 7.82E-05 | 2.193399458 |
| 343 | TAS2R19 | -0.71111288 | 6.305513743 | -4.510929603 | 2.46E-05 | 8.08E-05 | 2.158095895 |
| 344 | BTLA | -0.7381033 | 7.478115757 | -4.484406366 | 2.72E-05 | 8.82E-05 | 2.065212996 |
| 345 | SH2D1B | -0.718704382 | 6.749840871 | -4.468970714 | 2.87E-05 | 9.29E-05 | 2.011298219 |
| 346 | CD3G | -0.609653589 | 9.497249157 | -4.46751761 | 2.89E-05 | 9.34E-05 | 2.006228046 |
| 347 | CEACAM4 | 0.672953699 | 7.298926971 | 4.433768208 | 3.27E-05 | 0.00010413 | 1.888729467 |
| 348 | FAM133B | -0.63872237 | 2.842416471 | -4.415986203 | 3.48E-05 | 0.000110122 | 1.827023192 |
| 349 | MMP9 | 0.87550937 | 6.680908657 | 4.38887654 | 3.84E-05 | 0.000120257 | 1.733219161 |
| 350 | ERV3-1 | -0.969964843 | 5.220575843 | -4.388560622 | 3.85E-05 | 0.000120351 | 1.732127971 |
| 351 | DMTN | 0.617653488 | 7.532534471 | 4.360317848 | 4.26E-05 | 0.000131964 | 1.634758027 |
| 352 | N6AMT1 | -0.632221176 | 4.922809214 | -4.356466876 | 4.32E-05 | 0.000133642 | 1.621509333 |
| 353 | LGALSL | 0.641530497 | 8.071105129 | 4.356029708 | 4.33E-05 | 0.00013378 | 1.620005748 |
| 354 | RALGPS2 | -0.763926327 | 6.8446754 | -4.352015494 | 4.39E-05 | 0.000135438 | 1.606203414 |
| 355 | CCR7 | -0.620772815 | 8.862843057 | -4.345446538 | 4.50E-05 | 0.000138385 | 1.583632762 |
| 356 | GPR15 | -0.816748239 | 6.415599257 | -4.235993871 | 6.66E-05 | 0.000196859 | 1.210489625 |
| 357 | PRH1 | -0.63470266 | 3.325733629 | -4.232055103 | 6.75E-05 | 0.00019936 | 1.197166283 |
| 358 | CHI3L1 | 0.937687726 | 6.449440714 | 4.136748387 | 9.45E-05 | 0.000269776 | 0.877050895 |
| 359 | CHORDC1 | -0.962723516 | 4.461055229 | -4.126955438 | 9.78E-05 | 0.000278242 | 0.844408667 |
| 360 | SDHC | -0.804438761 | 5.350622757 | -4.081672872 | 0.000114626 | 0.000321163 | 0.694088105 |
| 361 | LCN2 | 1.02162019 | 7.641810386 | 4.057294135 | 0.000124785 | 0.000346343 | 0.613584123 |
| 362 | ZNF429 | -0.590725112 | 5.515769071 | -4.033854346 | 0.000135365 | 0.000373173 | 0.536463547 |
| 363 | SLC25A37 | 0.860376742 | 8.647064414 | 4.013547937 | 0.000145224 | 0.000397351 | 0.46987813 |
| 364 | TAS2R50 | -0.734210923 | 4.6142268 | -3.975877475 | 0.000165371 | 0.000445579 | 0.346915731 |
| 365 | SMU1 | -0.708112935 | 6.175818586 | -3.910977112 | 0.00020653 | 0.000544251 | 0.136801936 |
| 366 | PHOSPHO1 | 0.766217483 | 6.215150657 | 3.855643991 | 0.000249222 | 0.000643504 | -0.040577618 |
| 367 | ADGRG3 | 0.748853149 | 6.393329171 | 3.819492636 | 0.000281547 | 0.000719711 | -0.155576778 |
| 368 | KLRF1 | -0.665369614 | 6.489498086 | -3.750963819 | 0.000354148 | 0.000884468 | -0.371606519 |
| 369 | SLC25A33 | -0.773720543 | 4.360684143 | -3.745477967 | 0.000360676 | 0.000898937 | -0.388787644 |
| 370 | DEFA4 | 1.119425227 | 6.940989186 | 3.709888247 | 0.000405896 | 0.001000878 | -0.499841526 |
| 371 | ZNF208 | -0.691540816 | 4.180644014 | -3.703017611 | 0.000415227 | 0.001021684 | -0.521198489 |
| 372 | TLR10 | -0.6035245 | 5.127569886 | -3.678131958 | 0.000450765 | 0.001099662 | -0.598330075 |
| 373 | BANK1 | -0.659859252 | 7.184970186 | -3.552374588 | 0.000679281 | 0.001589314 | -0.982651528 |
| 374 | CEACAM8 | 1.146220775 | 6.731413286 | 3.515735502 | 0.000764288 | 0.00176866 | -1.092876635 |
| 375 | LTF | 1.062404195 | 8.572335429 | 3.489773236 | 0.000830525 | 0.001902686 | -1.170495764 |
| 376 | MPO | 0.640709005 | 6.4271375 | 3.473488966 | 0.000874808 | 0.001994324 | -1.218973473 |
| 377 | SLPI | 0.97636086 | 5.954753657 | 3.440175026 | 0.000972454 | 0.0021976 | -1.317646616 |
| 378 | BPI | 1.057049511 | 7.337140771 | 3.434146987 | 0.000991189 | 0.002235227 | -1.335428834 |
| 379 | SLC4A1 | 0.999098704 | 7.190807643 | 3.398321044 | 0.001109768 | 0.002470216 | -1.440651787 |
| 380 | PROK2 | 0.635491595 | 8.673651629 | 3.397149641 | 0.001113862 | 0.002477979 | -1.4440789 |
| 381 | CYP4F3 | 0.986478804 | 5.641846386 | 3.343073384 | 0.001319184 | 0.002892355 | -1.601357615 |
| 382 | ALAS2 | 1.271565654 | 7.438520829 | 3.318585982 | 0.00142345 | 0.003099171 | -1.67197535 |
| 383 | DCAF12 | 0.627952514 | 8.596230186 | 3.313473828 | 0.001446175 | 0.003144655 | -1.686670188 |
| 384 | SDHD | -0.598057408 | 5.3795539 | -3.178302748 | 0.002186439 | 0.004550107 | -2.069138894 |
| 385 | JCHAIN | -0.840379521 | 9.138244171 | -3.122227589 | 0.002587345 | 0.005292577 | -2.224305405 |
| 386 | SELENBP1 | 0.838755602 | 6.648320971 | 3.08315577 | 0.002906177 | 0.005873829 | -2.331184062 |
| 387 | MMP8 | 1.24535068 | 5.545674757 | 3.053481759 | 0.003172386 | 0.006346252 | -2.411669128 |
| 388 | IL1R2 | 0.909855159 | 6.342840714 | 3.043614758 | 0.003265817 | 0.006516435 | -2.438299314 |
| 389 | IFI44 | -0.684746766 | 6.943698786 | -3.028885424 | 0.00341006 | 0.006779797 | -2.477929229 |
| 390 | RNASE3 | 0.923351147 | 6.955157914 | 3.006698488 | 0.003638546 | 0.007197354 | -2.537344327 |
| 391 | FCRL1 | -0.692557377 | 7.620862214 | -2.961123183 | 0.004153131 | 0.008108629 | -2.658329539 |
| 392 | MGAM | 0.770292385 | 6.417455614 | 2.834135712 | 0.005963471 | 0.011209287 | -2.987774191 |
| 393 | ABCA13 | 0.699413286 | 5.264875929 | 2.766401924 | 0.007202859 | 0.013288408 | -3.158797259 |
| 394 | LYPLA1 | -0.613203082 | 4.813286486 | -2.759406145 | 0.007343488 | 0.013510063 | -3.176271888 |
| 395 | EGR1 | 0.667692916 | 8.572225929 | 2.670146899 | 0.009372216 | 0.016881998 | -3.396082055 |
| 396 | OLFM4 | 1.013440425 | 5.073802543 | 2.644388132 | 0.010046083 | 0.018003169 | -3.458419865 |
| 397 | CEACAM6 | 0.792170341 | 4.948829743 | 2.630324988 | 0.010432301 | 0.01865436 | -3.492244422 |
| 398 | KCNJ15 | 0.599490721 | 6.068792414 | 2.624747335 | 0.010589181 | 0.018919102 | -3.505618705 |
| 399 | ANXA2P2 | -0.620208236 | 5.9053098 | -2.476428752 | 0.015629584 | 0.026934019 | -3.852606056 |
| 400 | ANXA3 | 0.748579675 | 5.330857014 | 2.423932635 | 0.017875441 | 0.030425151 | -3.971363145 |
| 401 | CA1 | 0.867476244 | 5.031737329 | 2.410097535 | 0.018513501 | 0.031386569 | -4.002302668 |
| 402 | AHSP | 0.642101948 | 4.591349057 | 2.202415812 | 0.030849837 | 0.049376866 | -4.448450461 |

**Table S2 The top 25 compounds selected from Enamine HTS and top 25 natural products selected from ZINC as potential drugs targeting LRPPRC**

| **Top 25 compounds selected from Enamine HTS** | | | | | |
| --- | --- | --- | --- | --- | --- |
| **ID** | **Affinity**  **(Kcal/mol)** | **Rank** | **Name from pubchem** | **Formula** | **Detailed Information** |
| Z109823102 | -10.8 | 1 | 4-oxo-3-{2-oxo-2-[2-(piperidine-1-carbonyl)-3,4-dihydro-2H-1,4-benzoxazin-4-yl]ethyl}-3,4-dihydrophthalazine-1-carboxamide | C25H25N5O5 | <https://enaminestore.com/catalog/Z109823102> |
| Z79383944 | -10.8 | 2 | 6-(3,4-dihydroquinolin-1(2H)-ylcarbonyl)-3-phenyl-3,4-dihydro-1H-isochromen-1-one | C25H21NO3 | <https://enaminestore.com/catalog/Z79383944> |
| Z18792881 | -10.2 | 3 | 2-(4-Methylpiperidin-1-yl)-2-oxoethyl 2-(6-methoxynaphthalen-2-yl)quinoline-4-carboxylate | C29H28N2O4 | <https://enaminestore.com/catalog/Z18792881> |
| Z31753778 | -10.1 | 4 | 2-oxo-N-(4-{[3-(trifluoromethyl)phenyl]sulfamoyl}phenyl)-2H-chromene-3-carboxamide | C23H15F3N2O5S | <https://enaminestore.com/catalog/Z31753778> |
| Z16009222 | -10 | 5 | 4-[(2H-1,3-benzodioxol-5-yl)methylidene]-2-{[2-(2,3-dihydro-1H-indol-1-yl)-2-oxoethyl]sulfanyl}-1-(4-methylphenyl)-4,5-dihydro-1H-imidazol-5-one | C28H23N3O4S | <https://enaminestore.com/catalog/Z16009222> |
| Z240482172 | -10 | 6 | N-[(1H-1,3-benzodiazol-5-yl)(phenyl)methyl]-4-oxo-2,3,4,5-tetrahydro-1,5-benzothiazepine-7-carboxamide | C24H20N4O2S | <https://enaminestore.com/catalog/Z240482172> |
| Z86229731 | -10 | 7 | 6-[4-(Diphenylmethyl)piperazin-1-yl]-2,4-dimethyl-2,3,4,5-tetrahydro-1,2,4-triazine-3,5-dione | C22H25N5O2 | <https://enaminestore.com/catalog/Z86229731> |
| Z13601329 | -9.8 | 8 | 2-{2,5-dioxo-3',4'-dihydro-2'H-spiro[imidazolidine-4,1'-naphthalene]-1-yl}-N-[4-(piperidine-1-sulfonyl)phenyl]acetamide | C25H28N4O5S | <https://enaminestore.com/catalog/Z13601329> |
| Z31596603 | -9.8 | 9 | N'-[2-(4-benzoylphenoxy)propanoyl]-4-oxo-3,4-dihydrophthalazine-1-carbohydrazide | C25H20N4O5 | <https://enaminestore.com/catalog/Z31596603> |
| Z44493477 | -9.8 | 10 | N-(2H-1,3-benzodioxol-5-yl)-2-[4-(diphenylmethyl)piperazin-1-yl]propanamide | C27H29N3O3 | <https://enaminestore.com/catalog/Z44493477> |
| Z1021134202 | -9.7 | 11 | N2,N6-bis(7-oxo-4,5,6,7-tetrahydro-1,3-benzothiazol-2-yl)pyridine-2,6-dicarboxamide | C21H17N5O4S2 | <https://enaminestore.com/catalog/Z1021134202> |
| Z1442387712 | -9.7 | 12 | N'-[2-(phenylsulfanyl)phenyl]-N-(4,5,6,7-tetrahydro-1H-indazol-5-yl)ethanediamide | C21H20N4O2S | <https://enaminestore.com/catalog/Z1442387712> |
| Z1444341712 | -9.7 | 13 | 2-[3-(2,4-Dioxo-1,2,3,4-tetrahydroquinazolin-1-yl)propanoyl]-1,2,3,4-tetrahydroisoquinoline-3-carboxylic acid | C21H19N3O5 | <https://enaminestore.com/catalog/Z1444341712> |
| Z19712687 | -9.7 | 14 | ({[1,1'-Biphenyl]-2-yl}carbamoyl)methyl 3-hydroxynaphthalene-2-carboxylate | C25H19NO4 | <https://enaminestore.com/catalog/Z19712687> |
| Z240682930 | -9.7 | 15 | 2-(1,2-benzoxazol-3-yl)-N-{3-[(phenylcarbamoyl)amino]phenyl}acetamide | C22H18N4O3 | <https://enaminestore.com/catalog/Z240682930> |
| Z368761872 | -9.7 | 16 | 6-chloro-7-{[2-oxo-2-(1,2,3,4-tetrahydroquinolin-1-yl)ethyl]amino}-3,4-dihydro-2H-1,4-benzoxazin-3-one | C19H18ClN3O3 | <https://enaminestore.com/catalog/Z368761872> |
| Z56849551 | -9.7 | 17 | N-{[5-(2,4-difluorobenzamido)-1,3,3-trimethylcyclohexyl]methyl}-2,4-difluorobenzamide | C24H26F4N2O2 | <https://enaminestore.com/catalog/Z56849551> |
| Z1128903520 | -9.6 | 18 | 1-oxo-N-[3-(5-oxo-4,5-dihydro-1,2,4-oxadiazol-3-yl)phenyl]-1,2-dihydroisoquinoline-3-carboxamide | C18H12N4O4 | <https://enaminestore.com/catalog/Z1128903520> |
| Z192642086 | -9.6 | 19 | 1-{4-[4-(2-Phenylethyl)piperazine-1-carbonyl]benzenesulfonyl}-1,2,3,4-tetrahydroquinoline | C28H31N3O3S | <https://enaminestore.com/catalog/Z192642086> |
| Z19357809 | -9.6 | 20 | 7-[2-(9H-fluoren-3-yl)-2-oxoethyl]-1,3-dimethyl-2,3,6,7-tetrahydro-1H-purine-2,6-dione | C22H18N4O3 | <https://enaminestore.com/catalog/Z19357809> |
| Z26445805 | -9.6 | 21 | N'-(3-methyl-2,3-dihydro-1,3-benzothiazol-2-ylidene)-4-{[(4-methyl-2-oxo-2H-chromen-7-yl)oxy]methyl}benzohydrazide | C26H21N3O4S | <https://enaminestore.com/catalog/Z26445805> |
| Z26715301 | -9.6 | 22 | 3-[4-(3-phenylprop-2-en-1-yl)piperazine-1-carbonyl]-6H,7H,8H,9H,10H,12H-azepino[2,1-b]quinazolin-12-one | C27H30N4O2 | <https://enaminestore.com/catalog/Z26715301> |
| Z33555755 | -9.6 | 23 | N'-{2-[(1,1-dioxo-1lambda6,2-benzothiazol-3-yl)amino]propanoyl}-3-methyl-4-oxo-3,4-dihydrophthalazine-1-carbohydrazide | C20H18N6O5S | <https://enaminestore.com/catalog/Z33555755> |
| Z353295878 | -9.6 | 24 | 1-(Diphenylmethyl)-3-{[2-(morpholin-4-yl)pyridin-3-yl]methyl}urea | C24H26N4O2 | <https://enaminestore.com/catalog/Z353295878> |
| Z73344535 | -9.6 | 25 | 2-{2,5-dioxo-3',4'-dihydro-2'H-spiro[imidazolidine-4,1'-naphthalene]-1-yl}-N-{3-[2-(pyridin-2-yl)ethenyl]phenyl}acetamide | C27H24N4O3 | <https://enaminestore.com/catalog/Z73344535> |
| **Top 25 natural products selected from ZINC** | | | | | |
| **ID** | **Affinity**  **(Kcal/mol)** | **Rank** | **Name from pubchem** | **Formula** | **Molecule Information** |
| ZINC68568380 | -11.7 | 1 | (15R,16R)-6-[(15S,16S)-15,16-dimethyl-6-tetracyclo[6.6.2.04,15.011,16]hexadeca-1(14),2,4,6,8,10,12-heptaenyl]-15,16-dimethyltetracyclo[6.6.2.04,15.011,16]hexadeca-1(14),2,4,6,8,10,12-heptaene | C36H30 | <https://zinc.docking.org/substances/ZINC000068568380/> |
| ZINC68563949 | -11.5 | 2 | - | C40H40ClN2+ | <https://zinc.docking.org/substances/ZINC000068563949/> |
| ZINC70706523 | -11.5 | 3 | N-[2-(3-chlorophenyl)ethyl]-2-[(10S,15S)-10-(4-fluorophenyl)-12,14-dioxo-8,11,13-triazatetracyclo[7.7.0.02,7.011,15]hexadeca-1(9),2,4,6-tetraen-13-yl]benzamide | C34H26ClFN4O3 | <https://zinc.docking.org/substances/ZINC000070706523/> |
| ZINC85907291 | -11.5 | 4 | - | C59H64N3O+ | <https://zinc.docking.org/substances/ZINC000085907291/> |
| ZINC70706097 | -11.4 | 5 | 2-[(10S,15S)-10-(3-chlorophenyl)-12,14-dioxo-8,11,13-triazatetracyclo[7.7.0.02,7.011,15]hexadeca-1(9),2,4,6-tetraen-13-yl]-N-(4-ethylphenyl)benzamide | C34H27ClN4O3 | <https://zinc.docking.org/substances/ZINC000070706097/> |
| ZINC70704811 | -11.3 | 6 | 2-[(10S,15S)-10-(4-fluorophenyl)-12,14-dioxo-8,11,13-triazatetracyclo[7.7.0.02,7.011,15]hexadeca-1(9),2,4,6-tetraen-13-yl]-N-[(2S)-4-phenylbutan-2-yl]benzamide | C36H31FN4O3 | <https://zinc.docking.org/substances/ZINC000070704811/> |
| ZINC70704744 | -11.2 | 7 | N-[(4-chlorophenyl)methyl]-2-[(10S,15S)-10-(4-fluorophenyl)-12,14-dioxo-8,11,13-triazatetracyclo[7.7.0.02,7.011,15]hexadeca-1(9),2,4,6-tetraen-13-yl]benzamide | C33H24ClFN4O3 | <https://zinc.docking.org/substances/ZINC000070704744/> |
| ZINC08789284 | -11.1 | 8 | - | C31H44O4 | <https://zinc.docking.org/substances/ZINC000008789284/> |
| ZINC04281019 | -11 | 9 | - | C40H38O2 | <https://zinc.docking.org/substances/ZINC000004281019/> |
| ZINC70706188 | -10.9 | 10 | 2-[(10S,15S)-12,14-dioxo-10-phenyl-8,11,13-triazatetracyclo[7.7.0.02,7.011,15]hexadeca-1(9),2,4,6-tetraen-13-yl]-N-(4-ethylphenyl)benzamide | C34H28N4O3 | <https://zinc.docking.org/substances/ZINC000070706188/> |
| ZINC72330374 | -10.8 | 11 | 6-Hydroxy-5-[(6-methyl-4-oxo-3-oxa-13-azoniatetracyclo[7.7.1.02,7.013,17]heptadeca-1,6,8,13(17)-tetraen-5-ylidene)methyl]-1-[3-(trifluoromethyl)phenyl]pyrimidine-2,4-dione | C28H23F3N3O5+ | <https://zinc.docking.org/substances/ZINC000072330374/> |
| ZINC68565050 | -10.7 | 12 | 1-Phenylpentacyclo[6.6.6.02,7.09,14.015,20]icosa-2,4,6,9,11,13,15,17,19-nonaene | C26H18 | <https://zinc.docking.org/substances/ZINC000068565050/> |
| ZINC68582824 | -10.5 | 13 | (2S,6R,8R,12S)-4,10-bis(3-nitrophenyl)-1-phenyl-4,10-diazatetracyclo[5.5.2.02,6.08,12]tetradec-13-ene-3,5,9,11-tetrone | C30H20N4O8 | <https://zinc.docking.org/substances/ZINC000068582824/> |
| ZINC79189073 | -10.5 | 14 | 1-cyclooctyl-7-hydroxy-4-(quinolin-8-yl)-1H-benzofuro[3,2-b]pyrazolo[4,3-e]pyridin-3(2H)-one | C29H26N4O3 | <https://zinc.docking.org/substances/ZINC000079189073/> |
| ZINC02097246 | -10.4 | 15 | - | C32H27N3O3 | <https://zinc.docking.org/substances/ZINC000002097246/> |
| ZINC02125217 | -10.4 | 16 | 3-(4-bromo-3-methylphenyl)-3,4,7,8,9,10-hexahydro-2H,6H-benzo[3,4]chromeno[8,7-e][1,3]oxazin-6-one | C22H16BrNO3 | <https://zinc.docking.org/substances/ZINC000002125217/> |
| ZINC04270981 | -10.4 | 17 | N-[(1R,9S)-11-(naphthalene-2-carbonyl)-6-oxo-7,11-diazatricyclo[7.3.1.02,7]trideca-2,4-dien-5-yl]benzamide | C29H25N3O3 | <https://zinc.docking.org/substances/ZINC000004270981/> |
| ZINC12874427 | -10.4 | 18 | (2S,9R)-9-(2-chlorophenyl)-4-[2-(1H-indol-3-yl)ethyl]-2-methyl-4,7,17-triazatetracyclo[8.7.0.02,7.011,16]heptadeca-1(10),11,13,15-tetraene-3,6-dione | C31H27ClN4O2 | <https://zinc.docking.org/substances/ZINC000012874427/> |
| ZINC68587242 | -10.4 | 19 | - | C25H17Cl2NO2 | <https://zinc.docking.org/substances/ZINC000068587242/> |
| ZINC08790904 | -10.3 | 20 | - | C31H29FN4O3 | <https://zinc.docking.org/substances/ZINC000008790904/> |
| ZINC15959874 | -10.3 | 21 | (1R,3S,3aR,6aS)-1-[(3,4-dihydroxyphenyl)methyl]-5-(2-phenylethyl)spiro[1,2,3a,6a-tetrahydropyrrolo[3,4-c]pyrrole-3,3'-1H-indole]-2',4,6-trione | C28H25N3O5 | <https://zinc.docking.org/substances/ZINC000015959874/> |
| ZINC79204189 | -10.3 | 22 | (3beta)-cholest-5-en-3-yl [(3-methyl-6-oxo-7,8,9,10-tetrahydro-6H-benzo[c]chromen-1-yl)oxy]acetate | C43H60O5 | <https://zinc.docking.org/substances/ZINC000079204189/> |
| ZINC15675542 | -10.2 | 23 | 5-[(6S)-5-(1-benzofuran-2-ylmethyl)-3,4,6,7-tetrahydroimidazo[4,5-c]pyridin-6-yl]-3-[4-(trifluoromethyl)phenyl]-1,2,4-oxadiazole | C24H18F3N5O2 | <https://zinc.docking.org/substances/ZINC000015675542/> |
| ZINC68601323 | -10.2 | 24 | - | C30H19NO4 | <https://zinc.docking.org/substances/ZINC000068601323/> |
| ZINC70686494 | -10.2 | 25 | (2S,8S)-6-(1-benzylpiperidin-4-yl)-2-naphthalen-1-yl-3,6,17-triazatetracyclo[8.7.0.03,8.011,16]heptadeca-1(10),11,13,15-tetraene-4,7-dione | C36H34N4O2 | <https://zinc.docking.org/substances/ZINC000070686494/> |

**Table S3 The top 25 compounds selected from Enamine HTS and top 25 natural products selected from ZINC as potential drugs targeting FTO**

| **Top 25 compounds selected from Enamine HTS** | | | | | |
| --- | --- | --- | --- | --- | --- |
| **ID** | **Affinity (Kcal/mol)** | **Rank** | **Name from pubchem** | **Formula** | **Detailed Information** |
| Z28140847 | -10.2 | 1 | 3-benzyl-N-(3-chlorophenyl)-4-oxo-3,4-dihydrophthalazine-1-carboxamide | C22H16ClN3O2 | <https://enaminestore.com/catalog/Z28140847> |
| Z316147040 | -10.1 | 2 | N-{[4-(piperidine-1-carbonyl)phenyl]methyl}-2,3,4,9-tetrahydro-1H-carbazole-8-carboxamide | C26H29N3O2 | <https://enaminestore.com/catalog/Z316147040> |
| Z31323863 | -10 | 3 | N-(3-chlorophenyl)-N-(4-{[2-(3,4-dimethylphenyl)-5-oxo-4,5-dihydro-1,3-oxazol-4-ylidene]methyl}-1,3-thiazol-2-yl)acetamide | C23H18ClN3O3S | <https://enaminestore.com/catalog/Z31323863> |
| Z335602852 | -9.9 | 4 | 5-methyl-3-({9-methyl-4-oxo-4H-pyrido[1,2-a]pyrimidin-2-yl}methyl)-5-(naphthalen-1-yl)imidazolidine-2,4-dione | C24H20N4O3 | <https://enaminestore.com/catalog/Z335602852> |
| Z45588056 | -9.7 | 5 | N-(2H-1,3-benzodioxol-5-yl)-2-(2,6-difluorobenzenesulfonamido)benzamide | C20H14F2N2O5S | <https://enaminestore.com/catalog/Z45588056> |
| Z26445805 | -9.6 | 6 | N'-(3-methyl-2,3-dihydro-1,3-benzothiazol-2-ylidene)-4-{[(4-methyl-2-oxo-2H-chromen-7-yl)oxy]methyl}benzohydrazide | C26H21N3O4S | <https://enaminestore.com/catalog/Z26445805> |
| Z30879401 | -9.6 | 7 | 2-[4-(2-Phenylquinoline-4-carbonyl)piperazin-1-yl]phenol | C26H23N3O2 | <https://enaminestore.com/catalog/Z30879401> |
| Z31309202 | -9.6 | 8 | N-(4-methoxyphenyl)-N-{4-[(5-oxo-2-phenyl-4,5-dihydro-1,3-oxazol-4-ylidene)methyl]-1,3-thiazol-2-yl}acetamide | C22H17N3O4S | <https://enaminestore.com/catalog/Z31309202> |
| Z109823102 | -9.5 | 9 | 4-oxo-3-{2-oxo-2-[2-(piperidine-1-carbonyl)-3,4-dihydro-2H-1,4-benzoxazin-4-yl]ethyl}-3,4-dihydrophthalazine-1-carboxamide | C25H25N5O5 | <https://enaminestore.com/catalog/Z109823102> |
| Z142675830 | -9.5 | 10 | 3-[(2H-1,3-benzodioxol-5-yl)sulfamoyl]-N-(2-oxo-2,3-dihydro-1H-1,3-benzodiazol-5-yl)benzamide | C21H16N4O6S | <https://enaminestore.com/catalog/Z142675830> |
| Z203879352 | -9.5 | 11 | N-({2-[(1H-imidazol-1-yl)methyl]phenyl}methyl)-1-oxo-3-phenyl-3,4-dihydro-1H-2-benzopyran-6-carboxamide | C27H23N3O3 | <https://enaminestore.com/catalog/Z203879352> |
| Z29617205 | -9.5 | 12 | N-[4-(2,5-difluorophenyl)-1,3-thiazol-2-yl]-11,11-dioxo-8,11lambda6-dithia-1,10-diazatricyclo[7.4.0.0^{2,7}]trideca-2(7),3,5,9-tetraene-5-carboxamide | C19H12F2N4O3S3 | <https://enaminestore.com/catalog/Z29617205> |
| Z1891749053 | -9.4 | 13 | 1-(6-Chloro-2-hydroxy-4-phenylquinolin-3-yl)-3-(3,4-dichlorophenyl)prop-2-en-1-one | C24H14Cl3NO2 | <https://enaminestore.com/catalog/Z1891749053> |
| Z27091657 | -9.4 | 14 | 3-(3,4-dimethoxyphenyl)-N-(2-{5-[(4-fluorophenyl)methylidene]-2,4-dioxo-1,3-thiazolidin-3-yl}ethyl)prop-2-enamide | C23H21FN2O5S | <https://enaminestore.com/catalog/Z27091657> |
| Z27695147 | -9.4 | 15 | N-[(2H-1,3-benzodioxol-5-yl)methyl]-4-(1,2,3,4-tetrahydroisoquinoline-2-sulfonyl)benzamide | C24H22N2O5S | <https://enaminestore.com/catalog/Z27695147> |
| Z437015200 | -9.4 | 16 | N-{[6-(azepan-1-yl)pyridin-3-yl]methyl}-4-methoxy-1-phenyl-1H-pyrazole-3-carboxamide | C23H27N5O2 | <https://enaminestore.com/catalog/Z437015200> |
| Z46533115 | -9.4 | 17 | 2-{2-[1-benzyl-3-(pyridin-3-yl)-1H-pyrazol-4-yl]ethenyl}quinoline-4-carboxylic acid | C27H20N4O2 | <https://enaminestore.com/catalog/Z46533115> |
| Z87671029 | -9.4 | 18 | 3-[2-(4-acetamidophenyl)acetamido]-N-[3-(trifluoromethyl)phenyl]benzamide | C24H20F3N3O3 | <https://enaminestore.com/catalog/Z87671029> |
| Z1713527635 | -9.3 | 19 | N-[2-(2,3-dihydro-1,4-benzodioxin-5-yl)-2-oxoethyl]-4-(4-fluorophenyl)-5-methylthiophene-2-carboxamide | C22H18FNO4S | <https://enaminestore.com/catalog/Z1713527635> |
| Z26402982 | -9.3 | 20 | 2-benzyl-N-(6-methyl-1,3-benzothiazol-2-yl)-1,3-dioxo-2,3-dihydro-1H-isoindole-5-carboxamide | C24H17N3O3S | <https://enaminestore.com/catalog/Z26402982> |
| Z28246473 | -9.3 | 21 | 2H-1,3-benzodioxol-5-yl 4-chloro-8,8-dioxo-8lambda6-thia-1,9-diazatricyclo[8.5.0.0^{2,7}]pentadeca-2,4,6,9-tetraene-5-carboxylate | C20H17ClN2O6S | <https://enaminestore.com/catalog/Z28246473> |
| Z28486623 | -9.3 | 22 | 2-[({5,7-dimethyl-[1,2,4]triazolo[1,5-a]pyrimidin-2-yl}sulfanyl)methyl]-N-(quinolin-8-yl)benzamide | C24H20N6OS | <https://enaminestore.com/catalog/Z28486623> |
| Z30829669 | -9.3 | 23 | N-(2-{[(3-chlorophenyl)methyl]carbamoyl}phenyl)-3-methylbenzamide | C22H19ClN2O2 | <https://enaminestore.com/catalog/Z30829669> |
| Z337572294 | -9.3 | 24 | 2-{5H,6H,7H-indeno[5,6-b]furan-3-yl}-N-({2-[(propan-2-yloxy)methyl]phenyl}methyl)acetamide | C24H27NO3 | <https://enaminestore.com/catalog/Z337572294> |
| Z346162112 | -9.3 | 25 | 3-(3,5-dimethyl-1,2-oxazol-4-yl)-N-[2-(1,2,3,4-tetrahydroisoquinolin-2-yl)phenyl]propanamide | C23H25N3O2 | <https://enaminestore.com/catalog/Z346162112> |
| **Top 25 natural products selected from ZINC** | | | | | |
| **ID** | **Affinity**  **(Kcal/mol)** | **Rank** | **Name from pubchem** | **Formula** | **Detailed Information** |
| ZINC03875800 | -12.3 | 1 | - | C34H16O2 | <https://zinc.docking.org/substances/ZINC000003875800/> |
| ZINC70665164 | -11.6 | 2 | (3S,8S,9S,10R,13S,14S,17R)-17-[(2Z)-5,5-diphenylpenta-2,4-dien-2-yl]-10,13-dimethyl-2,3,4,7,8,9,11,12,14,15,16,17-dodecahydro-1H-cyclopenta[a]phenanthren-3-ol | C36H44O | <https://zinc.docking.org/substances/ZINC000070665164/> |
| ZINC04404594 | -11.5 | 3 | - | C30H14O2 | <https://zinc.docking.org/substances/ZINC000004404594/> |
| ZINC68569433 | -11.5 | 4 | 2-[(Z)-2-phenanthren-3-ylethenyl]benzo[c]phenanthrene | C34H22 | <https://zinc.docking.org/substances/ZINC000068569433/> |
| ZINC05220992 | -11.4 | 5 | Benzo[lmn]diquinazolino[2,1-b:2',3'-i][3,8]phenanthroline-5,9,11,19-tetraone | C28H12N4O4 | <https://zinc.docking.org/substances/ZINC000005220992/> |
| ZINC04309761 | -11 | 6 | - | C28H12N2O2 | <https://zinc.docking.org/substances/ZINC000004309761/> |
| ZINC05596479 | -11 | 7 | - | C23H20N4 | <https://zinc.docking.org/substances/ZINC000005596479/> |
| ZINC03845298 | -10.9 | 8 | 3-Methyl-1-(3-methyl-9,10-dioxoanthracen-1-yl)anthracene-9,10-dione | C30H18O4 | <https://zinc.docking.org/substances/ZINC000003845298/> |
| ZINC08879777 | -10.9 | 9 | - | C28H19NO6 | <https://zinc.docking.org/substances/ZINC000008879777/> |
| ZINC08918028 | -10.9 | 10 | - | C28H30N4O3 | <https://zinc.docking.org/substances/ZINC000008918028/> |
| ZINC02105209 | -10.8 | 11 | - | C27H24N4O2 | <https://zinc.docking.org/substances/ZINC000002105209/> |
| ZINC03845263 | -10.8 | 12 | 2-(2-Oxoacenaphthylen-1-ylidene)naphtho[3,2-e][1]benzothiole-1,6,11-trione | C28H12O4S | <https://zinc.docking.org/substances/ZINC000003845263/> |
| ZINC68569292 | -10.8 | 13 | 2-[(E)-2-phenylethenyl]hexahelicene | C34H22 | <https://zinc.docking.org/substances/ZINC000068569292/> |
| ZINC85878218 | -10.8 | 14 | 13-{3-[4-(dibenzo[b,f][1,4]oxazepin-11-yl)piperazin-1-yl]-3-oxopropyl}-8,13-dihydroindolo[2',3':3,4]pyrido[2,1-b]quinazolin-5(7H)-one | C38H32N6O3 | <https://zinc.docking.org/substances/ZINC000085878218/> |
| ZINC05422381 | -10.7 | 15 | - | C25H13NO3 | <https://zinc.docking.org/substances/ZINC000005422381/> |
| ZINC08918054 | -10.7 | 16 | - | C28H23ClN4O3 | <https://zinc.docking.org/substances/ZINC000008918054/> |
| ZINC08918388 | -10.7 | 17 | - | C28H23ClN4O3 | <https://zinc.docking.org/substances/ZINC000008918388/> |
| ZINC08918423 | -10.7 | 18 | - | C31H30N4O3 | <https://zinc.docking.org/substances/ZINC000008918423/> |
| ZINC09184209 | -10.7 | 19 | - | C28H21NO4 | <https://zinc.docking.org/substances/ZINC000009184209/> |
| ZINC20610362 | -10.7 | 20 | 4-phenyl-9-[(1R)-1,2,3,4-tetrahydronaphthalen-1-yl]-8,10-dihydropyrano[2,3-f][1,3]benzoxazin-2-one | C27H23NO3 | <https://zinc.docking.org/substances/ZINC000020610362/> |
| ZINC70712700 | -10.7 | 21 | (4R)-N-[2-(5-chloro-1H-indol-3-yl)ethyl]-4-[(3R,5S,7S,8R,9S,10S,13R,14S,17R)-3,7-dihydroxy-10,13-dimethyl-2,3,4,5,6,7,8,9,11,12,14,15,16,17-tetradecahydro-1H-cyclopenta[a]phenanthren-17-yl]pentanamide | C34H49ClN2O3 | <https://zinc.docking.org/substances/ZINC000070712700/> |
| ZINC02113691 | -10.6 | 22 | - | C26H20ClN3O | <https://zinc.docking.org/substances/ZINC000002113691/> |
| ZINC12659865 | -10.6 | 23 | 1-hydroxy-2-{[(1R,5S)-8-oxo-1,5,6,8-tetrahydro-2H-1,5-methanopyrido[1,2-a][1,5]diazocin-3(4H)-yl]carbonyl}anthracene-9,10-dione | C26H20N2O5 | <https://zinc.docking.org/substances/ZINC000012659865/> |
| ZINC12902247 | -10.6 | 24 | 3-[(10aS)-1,3-dioxo-1,5,10,10a-tetrahydroimidazo[1,5-b]isoquinolin-2(3H)-yl]-N-[2-(1H-indol-3-yl)ethyl]benzamide | C28H24N4O3 | <https://zinc.docking.org/substances/ZINC000012902247/> |
| ZINC13406202 | -10.6 | 25 | - | C26H18N2O4 | <https://zinc.docking.org/substances/ZINC000013406202/> |

**Table S4 Molecular structure of the top 25 compounds selected from Enamine HTS and top 25 natural products selected from ZINC as potential drugs targeting LRPPRC**

| **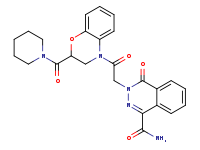         Z109823102** | **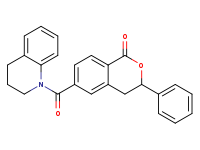         Z79383944** | **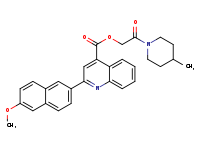         Z18792881** | **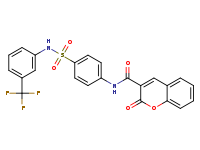         Z31753778** |
| --- | --- | --- | --- |
| **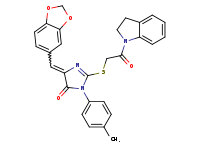         Z16009222** | **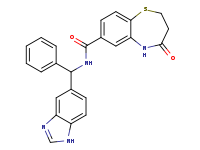         Z240482172** | **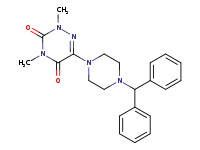         Z86229731** | **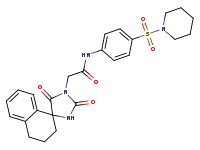         Z13601329** |
| **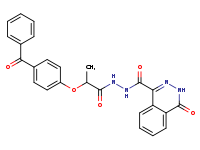         Z31596603** | **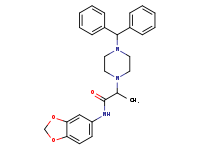         Z44493477** | **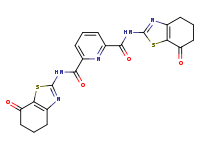         Z1021134202** | **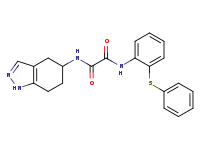         Z1442387712** |
| **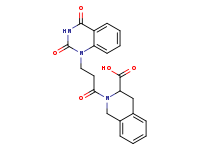         Z1444341712** | **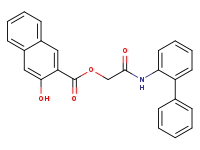         Z19712687** | **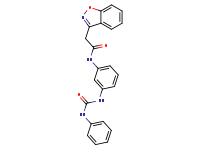         Z240682930** | **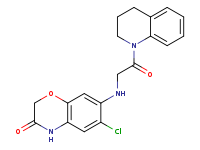         Z368761872** |
| **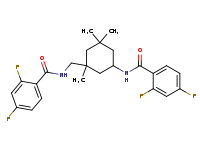         Z56849551** | **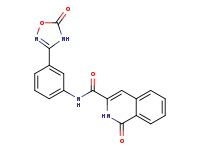         Z1128903520** | **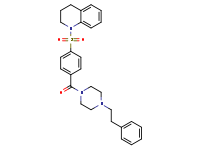         Z192642086** | **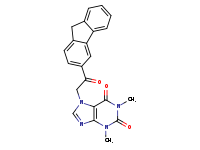         Z19357809** |
| **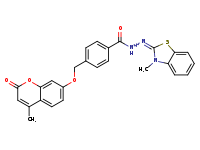         Z26445805** | **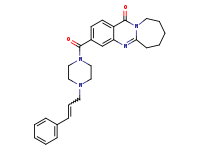         Z26715301** | **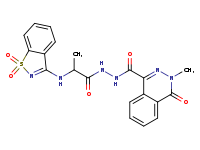         Z33555755** | **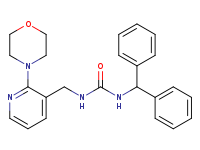         Z353295878** |
| **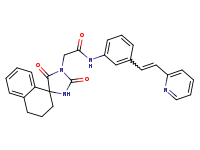         Z73344535** | **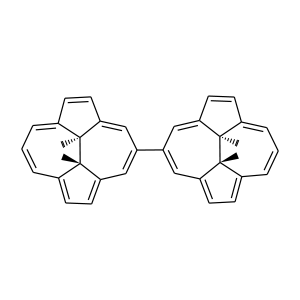         ZINC68568380** | **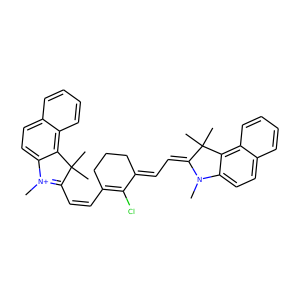         ZINC68563949** | **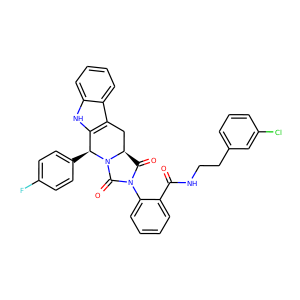         ZINC70706523** |
| **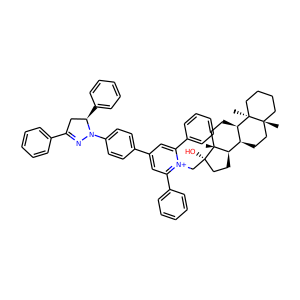         ZINC85907291** | **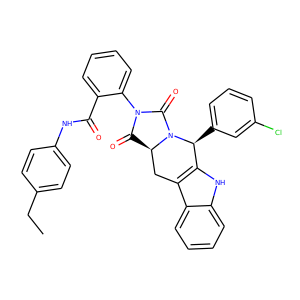         ZINC70706097** | **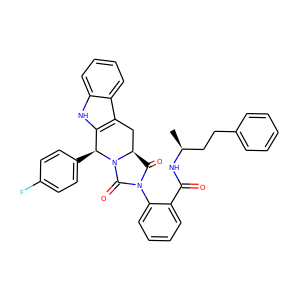         ZINC70704811** | **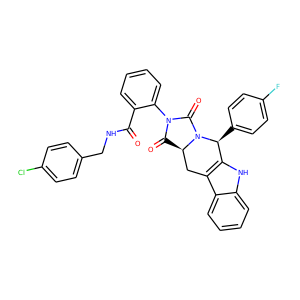         ZINC70704744** |
| **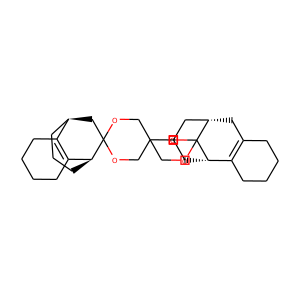         ZINC08789284** | **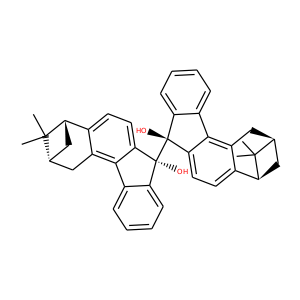         ZINC04281019** | **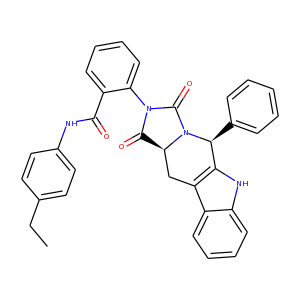         ZINC70706188** | **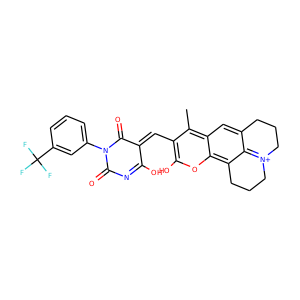         ZINC72330374** |
| **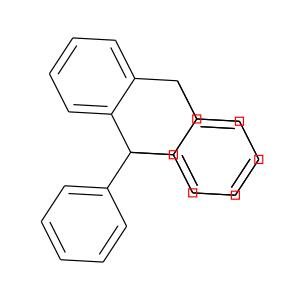         ZINC68565050** | **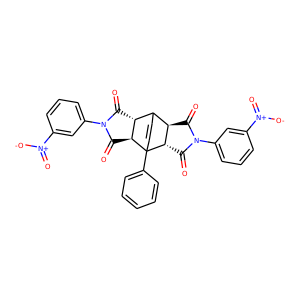         ZINC68582824** | **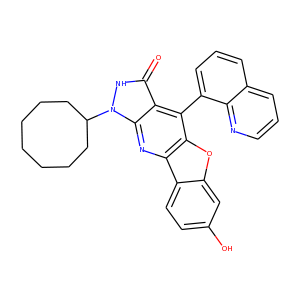         ZINC79189073** | **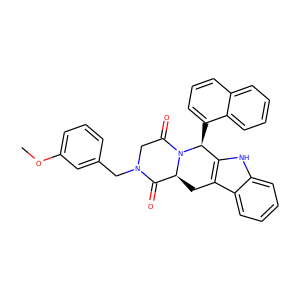         ZINC02097246** |
| **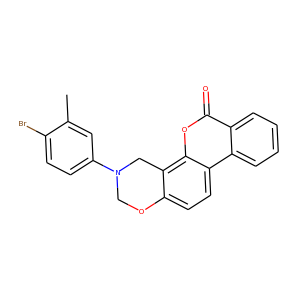         ZINC02125217** | **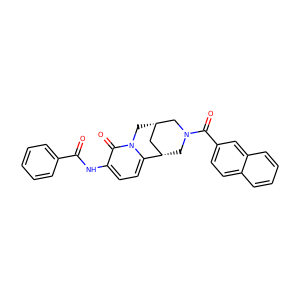         ZINC04270981** | **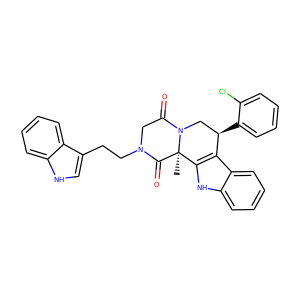         ZINC12874427** | **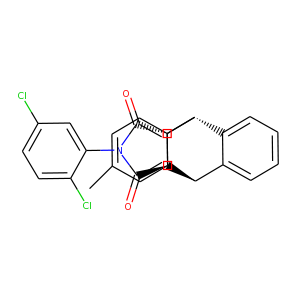         ZINC68587242** |
| **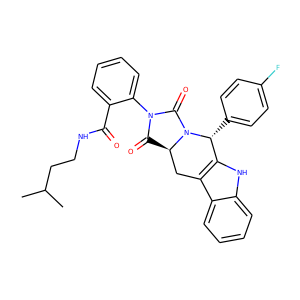         ZINC08790904** | **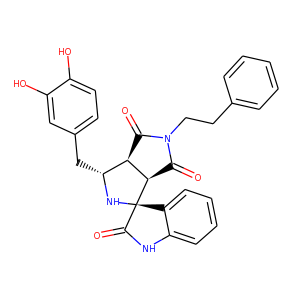         ZINC15959874** | **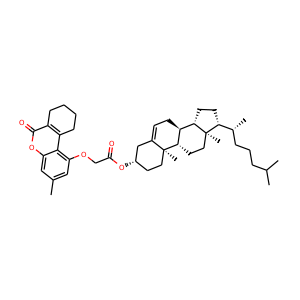         ZINC79204189** | **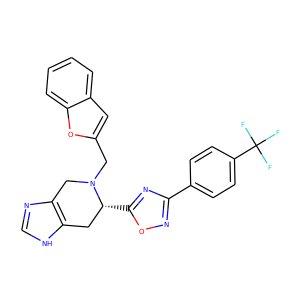         ZINC15675542** |
| **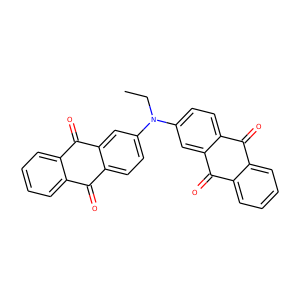         ZINC68601323** | **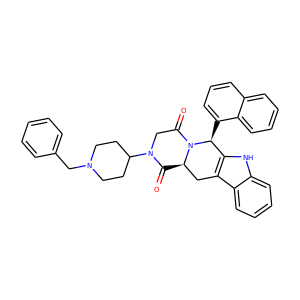         ZINC70686494** |  |  |

**Table S5 Molecular structure of the top 25 compounds selected from Enamine HTS and top 25 natural products selected from ZINC as potential drugs targeting FTO**

| **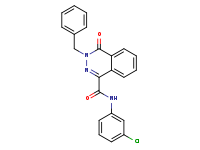         Z28140847** | **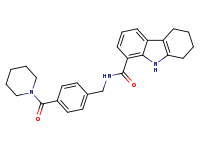         Z316147040** | **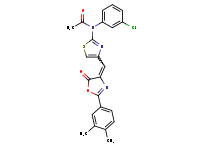         Z31323863** | **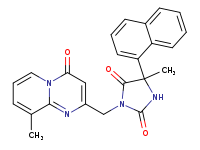         Z335602852** |
| --- | --- | --- | --- |
| **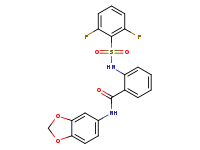         Z45588056** | **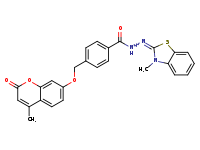         Z26445805** | **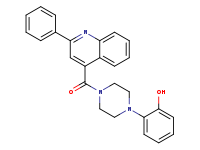         Z30879401** | **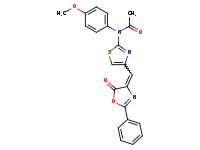         Z31309202** |
| **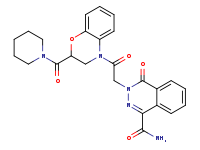         Z109823102** | **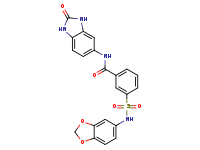         Z142675830** | **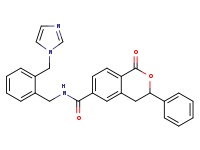         Z203879352** | **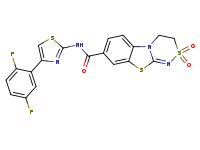         Z29617205** |
| **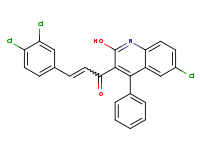         Z1891749053** | **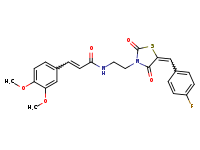         Z27091657** | **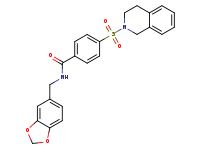         Z27695147** | **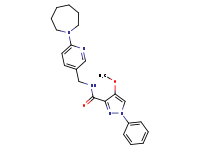         Z437015200** |
| **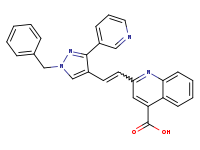         Z46533115** | **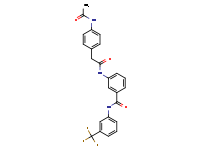         Z87671029** | **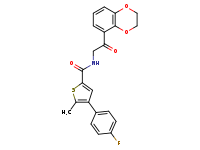         Z1713527635** | **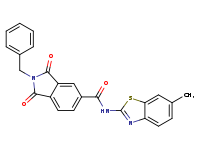         Z26402982** |
| **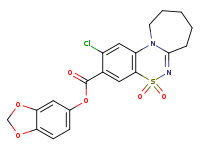         Z28246473** | **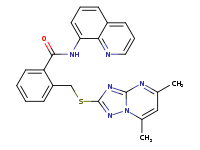         Z28486623** | **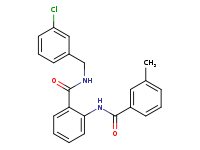         Z30829669** | **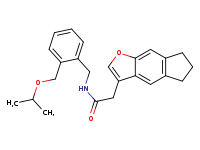         Z337572294** |
| **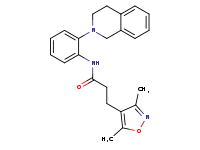         Z346162112** | **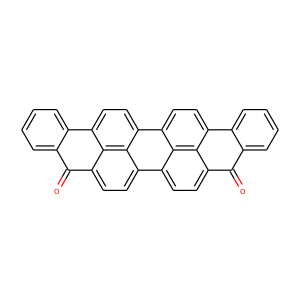         ZINC03875800** | **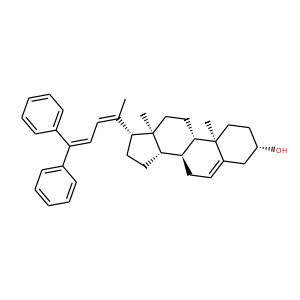         ZINC70665164** | **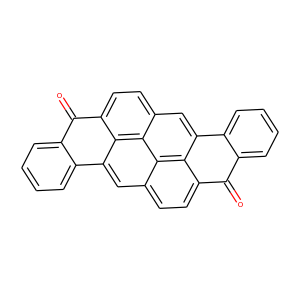         ZINC04404594** |
| **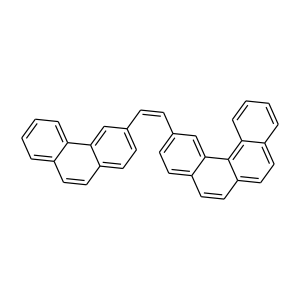         ZINC68569433** | **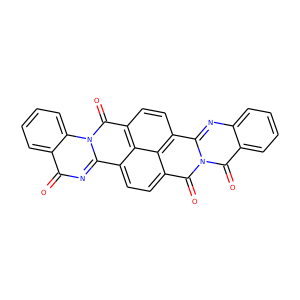         ZINC05220992** | **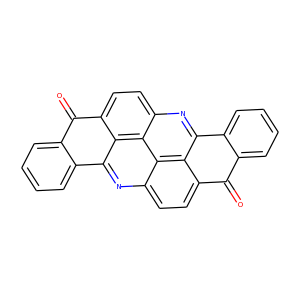         ZINC04309761** | **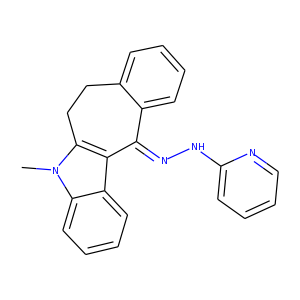         ZINC05596479** |
| **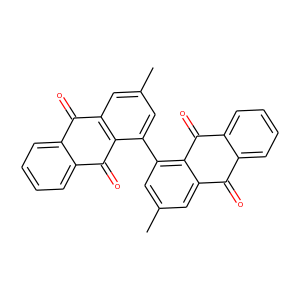         ZINC03845298** | **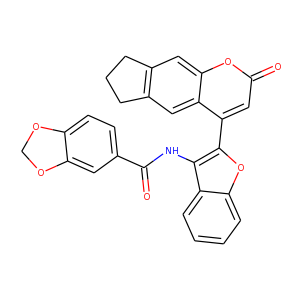         ZINC08879777** | **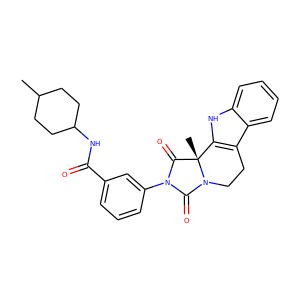         ZINC08918028** | **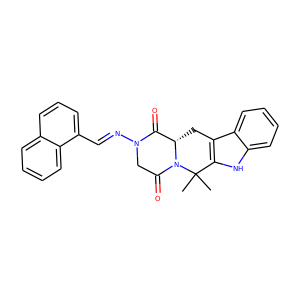         ZINC02105209** |
| **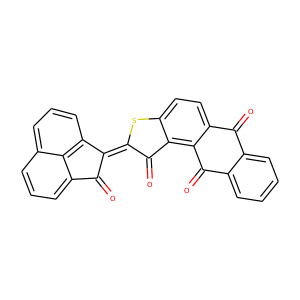         ZINC03845263** | **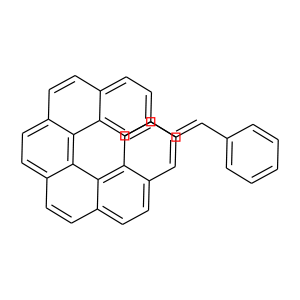         ZINC68569292** | **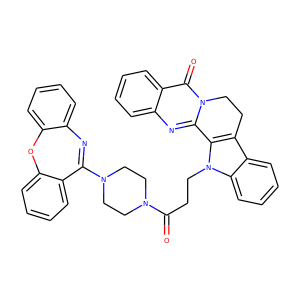         ZINC85878218** | **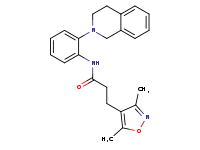         ZINC05422381** |
| **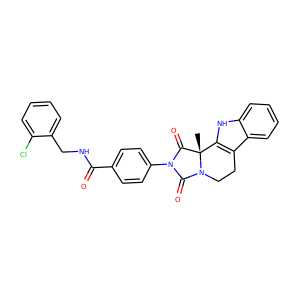         ZINC08918054** | **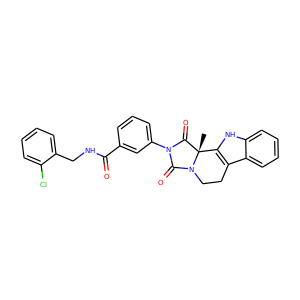         ZINC08918388** | **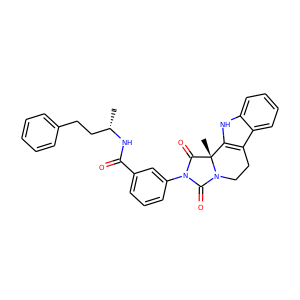         ZINC08918423** | **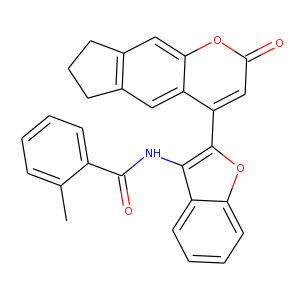         ZINC09184209** |
| **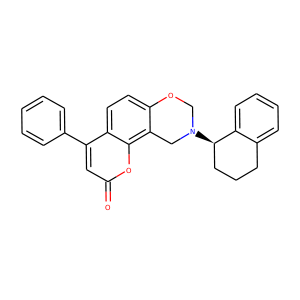         ZINC20610362** | **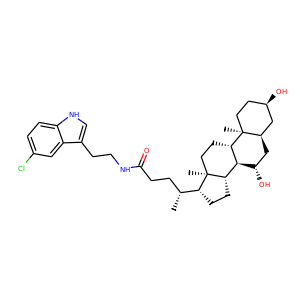         ZINC70712700** | **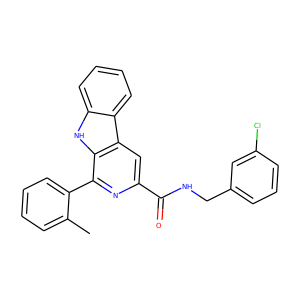         ZINC02113691** | **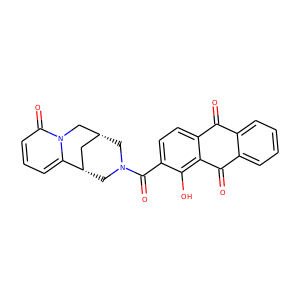         ZINC12659865** |
| **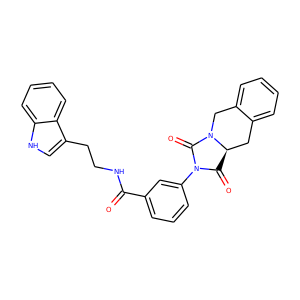         ZINC12902247** | **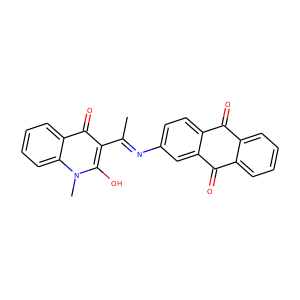         ZINC13406202** |  |  |
